# Stunting in infancy is associated with atypical activation of working memory and attention networks

**DOI:** 10.1038/s41562-023-01725-3

**Published:** 2023-10-26

**Authors:** Sobanawartiny Wijeakumar, Samuel H. Forbes, Vincent A. Magnotta, Sean Deoni, Kiara Jackson, Vinay P. Singh, Madhuri Tiwari, Aarti Kumar, John P. Spencer

**Affiliations:** 1 School of Psychology, University of Nottingham, Nottingham, UK; 2 Department of Psychology, Durham University, Durham, UK; 3 Department of Radiology, University of Iowa, Iowa City, IA USA; 4 Maternal, Newborn and Child Health Discovery & Tools, Bill & Melinda Gates Foundation, Seattle, WA USA; 5 Advanced Baby Imaging Lab, New England Pediatric Institute of Neurodevelopment, Rhode Island Hospital, Providence, RI USA; 6 School of Psychology, University of East Anglia, Norwich, UK; 7 Community Empowerment Lab, Lucknow, India

**Keywords:** Human behaviour, Developing world, Development of the nervous system

## Abstract

Stunting is associated with poor long-term cognitive, academic and economic outcomes, yet the mechanisms through which stunting impacts cognition in early development remain unknown. In a first-ever neuroimaging study conducted on infants from rural India, we demonstrate that stunting impacts a critical, early-developing cognitive system—visual working memory. Stunted infants showed poor visual working memory performance and were easily distractible. Poor performance was associated with reduced engagement of the left anterior intraparietal sulcus, a region involved in visual working memory maintenance and greater suppression in the right temporoparietal junction, a region involved in attentional shifting. When assessed one year later, stunted infants had lower problem-solving scores, while infants of normal height with greater left anterior intraparietal sulcus activation showed higher problem-solving scores. Finally, short-for-age infants with poor physical growth indices but good visual working memory performance showed more positive outcomes suggesting that intervention efforts should focus on improving working memory and reducing distractibility in infancy.

## Abstract (web summary)

Growth stunting is associated with poor long-term outcomes. Here Wijeakumar et al. show that, as early as infancy, stunting impairs visual working memory performance, suggesting a possible neurocognitive mechanism underlying this relationship.

## Main

Stunting or linear growth faltering often begins in utero and continues to unfold within the first 1,000 days of a child’s life. It is caused by a combination of factors such as poor nutrition, inadequate maternal health, exposure to infectious diseases and unhygienic environments, caregiver neglect and lack of stimulation. Stunting impacts ~150 million children under the age of 5 years worldwide^1^. In developing countries, stunting is associated with late enrolment in school and reduced educational attainment^2,3^. These deleterious effects typically continue into adulthood leading to a 20% reduction in adult income^4^, 1.4% loss in economic productivity^5^ and a total economic cost of $176.8 million per birth cohort^6^. Thus, stunting has an adverse impact at the individual, household and community levels, eventually perpetuating an intergenerational cycle of poverty and undernutrition.

Persistent stunting that begins early in life has a particularly strong impact on cognitive outcomes in later childhood. In a meta-analytic study conducted across 29 low-to-middle-income countries, a one-unit increase in height-adjusted *z*-scores (HAZ) for children under 2 years of age was associated with a 0.22 s.d. increase in cognition between 5 and 11 years of age^7^. Given this early impact, it is critical to understand how stunting affects neurocognitive mechanisms in the first year of life. Using portable neuroimaging techniques very early in development may be a powerful approach to address this issue. To date, only one neuroimaging study has investigated the impact of stunting on brain function in infancy^8^. This study examined growth measures and brain functional connectivity using electroencephalography in two groups of urban Bangladeshi children: a younger cohort aged 6 months and an older cohort aged 36 months. In the older cohort, there was an association between lower HAZ scores, greater brain functional connectivity in the theta and beta frequency bands and a reduction in children’s intelligence quotient scores at 48 months of age. Notably, the study did not find a link between HAZ scores and brain function in the younger 6-month-old cohort, nor did the study clarify how stunting might impact cognitive functions in the first year of life.

What cognitive systems are likely to be impacted by stunting in early development? One potential target is visual working memory (VWM), a critical cognitive system that emerges within months after birth, develops rapidly across early childhood and is susceptible to early risk factors^9^. Early in development, rich explorative play can enhance VWM and attentional networks leading to visual familiarity with objects and better retention of object-label associations which is important for word-learning and vocabulary development^10^. VWM processing is also predictive of individual differences in global fluid intelligence^11^ and academic outcomes^12^. For instance, WM processing has been linked to vocabulary scores^13^, non-symbolic scores^13^, comprehension^14^ and mathematics abilities^15^ in primary school and later school years. Given these predictive associations between VWM processing and later academic achievements and the insidious impact of stunting on cognitive and academic outcomes, it is important to examine VWM processing in infants at risk of stunted development.

**Fig. 1:**
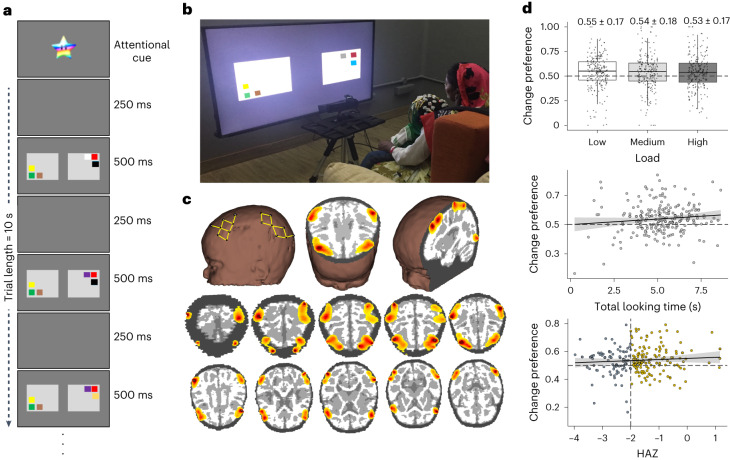
Experimental task and behavioural results. **a**, A trial of length 10 s of the preferential-looking VWM task. During the 10 s trial, alternating blank displays for 250 ms are followed by ‘on’ displays of coloured squares for 500 ms (250, 500, 250 and 500 ms… for 10 s). **b**, Exemplar of infant engaging with the task and wearing the fNIRS cap, sitting on the mother’s lap. **c**, Top left image shows probe geometry covering the frontal, temporal and parietal cortices. Top middle and right panels show sagittal and axial slices of photo migration results. Bottom panel shows superior (top) to inferior (bottom) axial slices of photon migration results. Redder colours indicate stronger signal sensitivity. **d**, Main effects from the analysis of CP scores showing modulation over memory load (box plot in top panel shows median with lower and upper hinges corresponding to the first and third quartiles and upper/lower whiskers extends from the upper/lower hinges to largest/smallest value or 1.5× interquartile range), TLT (scatter plot in middle panel) and HAZ (scatter plot in lower panel). In scatterplots, 0.95 CI is shown in grey. Colours in lower panel reflect typical cut-off scores used to identify stunted individuals (*z*-scores < −2) with grey showing stunted infants. *n* = 223 6- and 9-month-old infants.

How might VWM be assessed in infancy? One option is to use a preferential-looking task to measure looking behaviour and VWM function^16–18^. In this task, infants are presented with two flashing side-by-side displays of coloured squares (Fig. 1a,b). On the ‘unchanging’ side, the colours of the squares remain the same after each flash, while on the ‘changing’ side, there is a change in the colour of one square after each flash. If infants can maintain the colours on the unchanging display in VWM during the delay, they should lose interest in this display, releasing fixation to visually explore the ‘changing’ display. Here, they should detect the change and sustain looking to this display, leading to a strong change-preference (CP) score—a high proportion of looking to the changing side.

Previous work has shown that CP scores vary with the number of presented items (VWM load)^16,19,20^ and there is a developmental improvement in VWM capacity with age. Others have shown that 6.5-month-old infants demonstrate greater-than-chance CP scores with a VWM load of one item, while older infants of 10 and 13 months of age demonstrate greater-than-chance CP scores for VWM loads of two and three items^16^. Similarly, both 6- and 8-month-old infants show a preference for one complex object but only 8-month-old infants display a preference for two complex objects^19^.

Critically, recent neuroimaging work has shown that infants engaging with the preferential-looking VWM task modulate a frontotemporoparietal VWM network typically activated in adults. This work used functional near-infrared spectroscopy (fNIRS) to measure brain function in urban US infants^17^. Results revealed that visual exploratory measures were associated with activation of the dorsolateral prefrontal cortex (DLPFC). Further, infants showed task-dependent modulation of the anterior intraparietal sulcus (aIPS) and the temporoparietal junction (TPJ)^21,22^. In adult studies, aIPS, a part of the dorsal attention network, is associated with maintaining working memory representations^23,24^, while TPJ, a part of the ventral attention network, is typically suppressed in working memory tasks reflecting suppression of attentional shifts away from task goals^21^.

In the current study, we used the same VWM task and fNIRS neuroimaging methods to conduct the first-ever community-based study investigating the impact of stunting on neurocognitive function in infants in rural India. We situated the study in a low-resource setting in Uttar Pradesh; a recent initiative investigating child growth failures under the National Nutrition Mission reported that 97.3% of the state’s districts fell within the high tertile for stunting^25^. Our motivation for choosing this location was further justified by findings in a sample of children in Shivgarh, Uttar Pradesh, India: children from lower socioeconomic status families showed poorer VWM performance and distractor suppression in inferior frontal gyrus (IFG), a region in the VWM network^18^. Thus, in the current study, we recruited 6- and 9-month-old infants from high and low socioeconomic families and followed them for 2 years. We included both ages to assess whether this cohort would demonstrate shifts in behaviour between the ages of 6 and 9 months in line with previous work^19,20^. Alternatively, studying these two age groups might reveal delays in behaviour and/or brain function relative to urban US infants. Portable eye-tracking and video recordings were used to examine how infants visually explored the VWM task. The fNIRS was used to measure brain function in the infants as they engaged with the task (Fig. 1c). Growth measures were collected at multiple time-points across the 2 years to determine stunting status and growth rate. Finally, the ages-and-stages questionnaire (ASQ) was administered in the second year to examine the impact of stunting and VWM function on later cognitive outcomes.

We focused on three central questions. First, we inquired whether stunting was associated with poorer VWM performance in the first year of life. Second, we asked how stunting impacted brain function—do stunted infants differentially engage the frontoparietal VWM network compared to normal height infants? Finally, we investigated whether VWM performance and brain function in the first year were related to cognitive outcomes a year later and how these outcomes were modulated by stunting.

## Results

### Impact of stunting on VWM behaviour in infancy

We asked whether stunting was associated with poorer VWM performance in the first year of life. To address this question, we analysed CP scores using a linear model with age (6 and 9 months), load (one, two or three coloured items on each display), HAZ score and total looking time (TLT) as predictors. We included TLT in the model because it was not correlated with CP and captured a substantial proportion of variance in the CP scores. Furthermore, CP scores were calculated over a critical time window (Methods) while TLT was calculated across the full duration of the trial. All interactions among predictor variables were included. There were significant main effects of load (*F*(1,639) = 6.14, *P* = 0.013, effect size *η*_p_^2^ = 0.009, confidence interval (CI) = 0.03–0.23), TLT (*F*(1,639) = 6.86, *P* = 0.009, *η*_p_^2^ = 0.01, CI = 0.01–0.10) and HAZ (*F*(1,639) = 5.62, *P* = 0.018, *η*_p_^2^ = 0.008, CI = −0.24 to −0.02) on CP scores (Fig. 1d). As in previous studies conducted with Western samples, CP scores declined as the load increased. Further, infants with higher CP scores explored the displays for longer durations. Critically, a decrease in HAZ scores was associated with lower CP scores, reflecting poorer VWM performance in infancy.

There were also significant two-way interactions between HAZ and load (*F*(1,639) = 5.42, *P* = 0.02, *η*_p_^2^ = 0.008, CI = 0.01–0.11), load and TLT (*F*(1,639) = 6.30, *P* = 0.012, *η*_p_^2^ = 0.009, CI = −0.04 to −0.01) and HAZ and TLT (*F*(1,639) = 5.95, *P* = 0.015, *η*_p_^2^ = 0.009, CI = 0.01–0.05); these interactions were subsumed by a significant three-way interaction between HAZ, load and TLT (*F*(1,639) = 5.43, *P* = 0.02, *η*_p_^2^ = 0.008, CI = −0.02 to −0.00). Follow-up tests revealed a robust interaction between HAZ and TLT at the low load (*F*(1,211) = 6.68, *P* = 0.01, *η*_p_^2^ = 0.03, CI = 0.00–0.03) with no significant interactions at the medium and high loads (all *P* > 0.05). At the low load, increasing HAZ scores were associated with increasing CP scores in infants with longer looking durations (Supplementary Fig. 1a). By contrast, infants who failed to sustain longer looking durations had difficulty detecting the changing side with CP scores near chance levels (that is, 0.50).

Finally, there was a significant two-way interaction between age and HAZ (*F*(1,639) = 4.24, *P* = 0.04; *η*_p_^2^ = 0.006, CI = −0.45 to −0.01; Supplementary Fig. 1b). This effect appeared to be more strongly driven by 6-month-olds compared to 9-month-olds. Specifically, 6-month-olds showed a strong linear relationship between CP scores and HAZ scores, with infants of normal height showing higher CP scores. By contrast, 9-month-old infants did not demonstrate a linear relationship between CP scores and HAZ scores. They did better in the task overall with some infants showing CP scores that were greater than chance.

### VWM brain network engaged in infants from rural India

Our next goal was to examine infants’ brain function as they engaged in the VWM task. Coregistered beta maps from individual-level general linear modelling (Methods) were entered into a group-level linear mixed effects model with load (1, 2 and 3), chromophore (HbO and HbR), CP scores, age and HAZ scores as predictors. The model also included a random intercept for each participant to account for individual-level variance. We focused on significant main effects and interactions that included chromophore, as HbO and HbR are typically anticorrelated. Group-level clusters with significant effects following family-wise correction (Methods) are shown in Table 1. We discuss each set of effects below.

**Table 1:** Significant clusters of brain function engaged during the preferential-looking task obtained from running a three-dimensional linear mixed effects model

Effect	Region of interest	Size (mm^3^)	Centre of mass coordinates		
			*x*	*y*	*z*
CP × chromophore	rIFG	424	46.3	−154.2	142.2
Chromophore	laIPS	626	144.7	−103.8	181.6
Age × chromophore	laIPS	400	138.1	−97	186.3
Age × CP × chromophore	laIPS	354	155.5	−87.5	169.9
HAZ × load × chromophore	laIPS	562	139.1	−91.3	182.7
HAZ × age × CP × chromophore	laIPS	328	143.2	−103.5	181
HAZ × CP × chromophore	rTPJ	541	44	−80.7	152.1
HAZ × age × chromophore	lDLPFC	593	127.6	−162.7	171.5
Load × CP × chromophore	rFEF	603	74.6	−146.6	183.5

Note that significance was determined using family-wise correction for multiple comparisons with voxel-wise *P* threshold of 0.01, alpha threshold of 0.05, 10,000 iterations, one-sided thresholding, NN = 1 and with a minimum cluster size of 278 voxels.

**Fig. 2:**
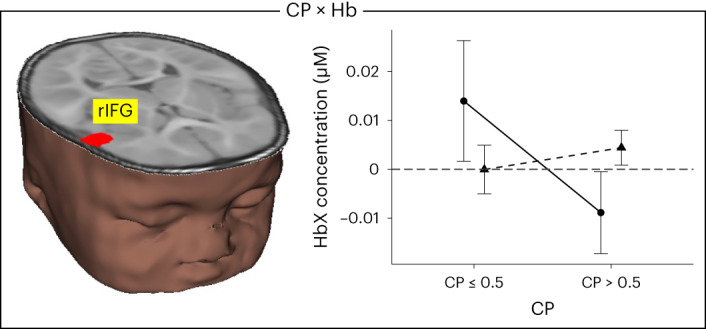
Interaction between CP score and chromophore. Brain image shows effect in rIFG (red cluster in brain inset). Right panel shows mean ± s.e. HbO (solid, circles) and HbR (dashed, triangles) concentrations across the haemodynamic time window (0–20 s) for infants with CP scores less than chance (≤0.5) and CP scores greater than chance. *n* = 221 6- and 9-month-old infants.

Infants engaged a canonical frontotemporoparietal VWM network while visually exploring the displays in the preferential-looking VWM task. Critically, we replicated a finding from our previous study in the same population: there was a significant interaction between CP score and chromophore in the right IFG (rIFG). As shown in Fig. 2, infants with weak or supressed activation in rIFG (lower HbO concentration/higher HbR concentration) showed higher CP scores. In previous work, greater IFG activation has been associated with poorer distractor suppression^18,26^. Thus, our findings suggest that infants with better VWM scores were able to suppress looks to the unchanging side via rIFG suppression. On the other hand, infants who activated rIFG showed poor distractor suppression, more frequent looks to the unchanging side and lower CP scores.

**Fig. 3:**
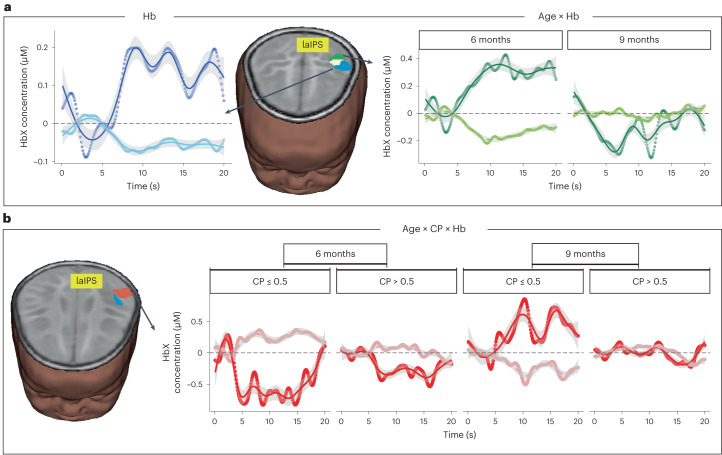
General effects in laIPS. **a**, Brain image shows location of main effect of chromophore (blue), interaction between age and chromophore (green) and overlap (white) in laIPS. Left panel: average time-series plots show HbO concentration in dark blue and HbR concentration in light blue for main effect of chromophore. Right panel: average time-series plots show HbO concentration in dark green and HbR concentration in light green for interaction between age and chromophore. We show data across the total haemodynamic time window (0–20 s) with 0 as trial onset and 10 s as trial offset. **b**, Brain image shows location of interaction between age, CP score and chromophore (red) close to the main effect of chromophore (blue). Right panel: average time-series plots showing HbO concentration in red and HbR concentration in light red for interaction between age, CP score and chromophore. We show data for 6- and 9-month-old infants with CP scores less than chance (≤0.5) and CP scores greater than chance. In time-series plots, the mean is depicted using circle data points; mean smoothed data using a LOESS function are shown as solid line. Confidence intervals are underlaid and shown in grey. *n* = 221 6- and 9-month-old infants.

One main effect and multiple interaction effects were observed in different clusters of the left anterior intraparietal sulcus (laIPS) (laIPS; Table 1), a part of the dorsal attention network engaged during VWM maintenance. We discuss the developmental and performance-related effects observed in laIPS first; stunting-related effects are discussed below. A main effect of chromophore was observed in the anterior portion of the laIPS, with the canonical pattern of increasing HbO and decreasing HbR concentrations (left panel in Fig. 3a and blue cluster on brain image). An interaction between age and chromophore was also observed in an adjoining laIPS cluster (right panels in Fig. 3a and green cluster on brain image). Specifically, 6-month-old infants showed greater laIPS activation compared to 9-month-old infants. Considered together with the main effect, the interaction between age and chromophore suggests refinement of laIPS activation with development.

This pattern was qualified by the significant interaction between age, CP score and chromophore in the inferior portion of the laIPS shown in Fig. 3b (red cluster). Here, 9-month-old infants who performed poorly in the VWM task showed engagement of laIPS, while higher-performing 9-month-olds did not. Low-performing 6-month-old infants, by contrast, showed suppression in this laIPS cluster. Thus, the overall pattern shown in Fig. 3 suggests that laIPS activation becomes more refined with age and enhanced performance in the task. There is also evidence that the youngest, low-performing infants showed a different pattern (suppression) in an inferior portion of the laIPS.

Before turning to the stunting-related effects, we note there was a significant interaction between load, CP score and chromophore in the right frontal eye fields (rFEF; Table 1). Activation in this region is associated with preparation and control of eye-movements and gaze^27,28^. We generally found suppression of rFEF for both higher- and lower-performing infants (Supplementary Fig. 2); however, this effect was inconsistent as a function of load. It is possible that the lack of a clear pattern with increasing load reflects ongoing functional refinement in this region relative to infants’ improving visual exploratory abilities.

### Impact of stunting on brain function

The findings above confirm that infants from rural India engaged a canonical VWM brain network and replicated previous findings from this population. Next, we asked how stunting impacted brain function in infancy. We found that stunting impacted activation in three regions (Table 1): (1) laIPS, a key region in the dorsal attention network, (2) right temporoparietal junction (rTPJ), a key region of the ventral attention network and (3) left dorsolateral prefrontal cortex (lDLPFC), an area involved in top-down control of processing in posterior regions of the brain.

**Fig. 4:**
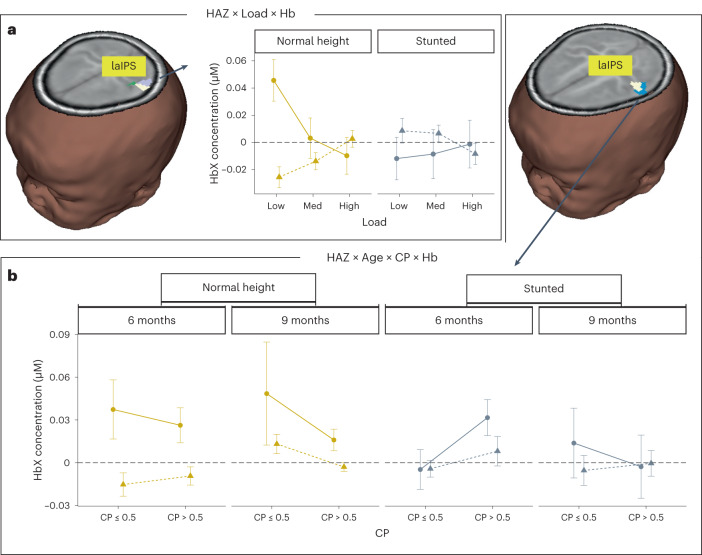
Stunting-related effects in laIPS. **a**, Brain image shows location of interaction between HAZ, load and chromophore (lavender), interaction between age and chromophore (green cluster from Fig. 3a) and overlap (white) in a superior cluster in laIPS. Right panel shows mean ± s.e. HbO (solid, circles) and HbR (dashed, triangles) concentration across the haemodynamic time window (0–20 s) for normal height (yellow) and stunted (grey) infants at the low, medium and high loads. **b**, Brain image shows location of interaction between HAZ, age, CP score and chromophore (white represents overlap between this interaction and main effect of chromophore) and main effect of chromophore (blue cluster from Fig. 3a) in a superior cluster in laIPS. Note that the cluster for the interaction between HAZ, age, CP score and chromophore was subsumed by the overlap (shown in white). Bottom panel shows mean ± s.e. HbO (solid, circles) and HbR (dashed, triangles) concentration across the haemodynamic time window (0–20 s) for normal height (yellow) and stunted (grey) 6- and 9-month-old infants with CP scores less than chance (≤0.5) and CP scores greater than chance. *n* = 221 6- and 9-month-old infants.

A significant three-way interaction between HAZ, load and chromophore was observed in a superior laIPS cluster (lavender cluster in Fig. 4a). Here, infants of normal height engaged laIPS in a load-dependent manner consistent with infants from an urban US sample with a decrease in activation at higher loads^17^. By contrast, stunted infants did not modulate laIPS activation with increasing load, although they showed some evidence of greater suppression of laIPS at the low load (negative HbO and positive HbR).

Activation in laIPS was also related to behavioural performance and stunting through a four-way interaction between HAZ, age, CP score and chromophore in a more inferior laIPS cluster (white cluster in Fig. 4b). Both 6- and 9-month-old infants of normal height showed robust activation in this laIPS cluster, with lower activation for higher-performing infants. This is consistent with results from Fig. 3 suggesting a refinement in laIPS activation with age and enhanced task performance. By contrast, stunted infants showed much weaker activation in this cluster. The one exception was 6-month-old stunted infants who performed better in the VWM task; these infants showed modest laIPS activation.

**Fig. 5:**
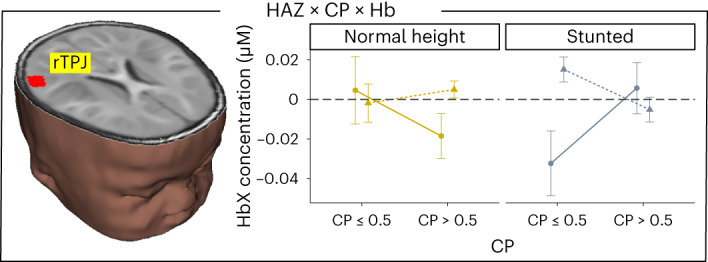
Stunting-related effect in rTPJ. Brain image shows location of interaction between HAZ, CP score and chromophore in rTPJ. Right panel shows mean ± s.e. HbO (solid, circles) and HbR (dashed, triangles) concentration across the haemodynamic time window (0–20 s) for normal height (yellow) and stunted (grey) infants with CP scores less than chance (≤0.5) and CP scores greater than chance. *n* = 221 6- and 9-month-old infants.

The next finding is shown in Fig. 5: suppression in rTPJ was related to behavioural performance through a three-way interaction between HAZ, CP score and chromophore. In infants of normal height, greater rTPJ suppression was associated with better CP scores. This finding is consistent with previous adult studies showing rTPJ suppression (along with laIPS activation) during VWM processing to prevent shifts in attention away from task goals^21^. In contrast, in stunted infants, greater rTPJ suppression was associated with poorer CP scores suggesting that stunted children tended to maintain attention to the unchanging side resulting in rTPJ suppression and poor CP scores.

**Fig. 6:**
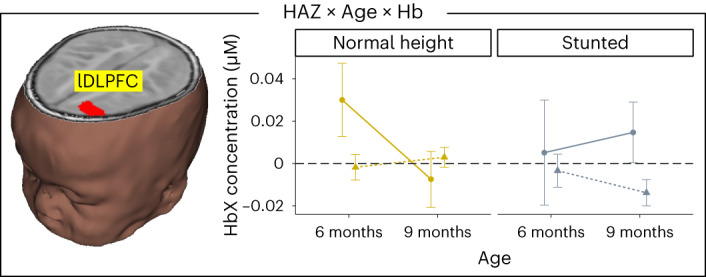
Stunting-related effect in lDLPFC. Brain image shows location of interaction between HAZ, age and chromophore in lDLPFC. Right panel shows mean ± s.e. HbO (solid, circles) and HbR (dashed, triangles) concentration across the haemodynamic time window (0–20 s) for 6- and 9-month-old normal height (yellow) and stunted (grey) infants. *n* = 221 6- and 9-month-old infants.

Finally, a three-way interaction between HAZ, age and chromophore was observed in lDLPFC (Fig. 6). This interaction was driven by increased lDLPFC activation in normal height 6-month-olds and stunted 9-month-olds. Evidence suggests that the DLPFC is often engaged in working memory tasks to support processing in the parietal cortex early in development and during demanding working memory tasks^29^. Our findings suggest, therefore, that normal height 6-month-olds and stunted 9-month-olds recruited this frontal area in support of their VWM performance. We note that this is the one brain region that showed robust activation in stunted 9-month-old infants, some of whom successfully detected the changing side. Thus, the delayed recruitment of this frontal region may be adaptive for these infants.

### Impact of stunting on longer-term cognitive outcomes

As a final question, we investigated whether stunting, behavioural performance and associated brain function in the first year of life were linked to cognitive outcomes 1 year later. In an initial linear model, we asked whether higher cognitive outcome scores in year 2 (problem-solving score from the ASQ; Methods) were associated with CP in year 1 and physical growth measures. For the physical growth measures, we included HAZ (the intercept from our growth model; Methods) and a linear growth term (HAZ-L), which captured change in HAZ over time, as predictors.

**Fig. 7:**
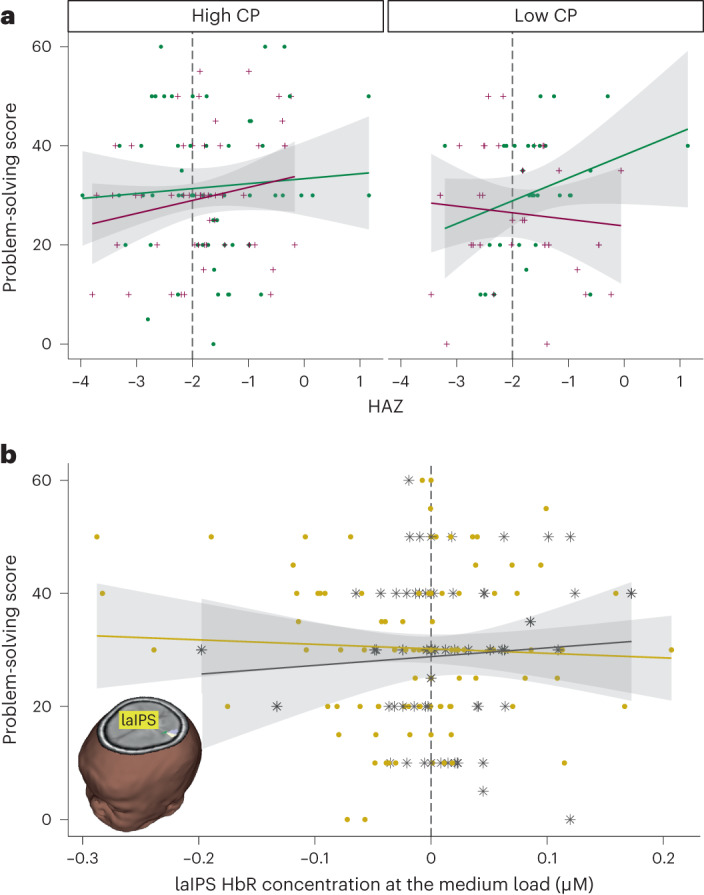
Impact on problem-solving outcomes. **a**, Impact of HAZ and CP scores on problem-solving outcomes 1 year later. Infants with higher linear growth from year 1 to year 2 based on a median split are shown as green circles; infants with lower linear growth are shown as purple '+'. Left panel shows scatter plot with infants with higher CP scores in year 1 based on a median split, while right panel shows scatter plot with data from infants with lower CP scores in year 1. Vertical line shows typical stunting cut-off value (HAZ < −2). **b**, Association between HAZ and laIPS HbR concentration from medium load in year 1 on problem-solving scores 1 year later. Infants of normal height (HAZ ≥ −2) are shown as yellow circles; stunted infants (HAZ < −2) are shown as grey asterisks. Inset brain image shows location of the laIPS cluster (from Fig. 4a). Across all plots, 0.95 CI is shown in grey.

Results revealed a significant interaction between HAZ and HAZ-L in predicting problem-solving scores in year 2 (*F*(1,164) = 4.30, *P* = 0.04, *η*_p_^2^ = 0.03, CI = 0.07–3.05). This effect was subsumed by a significant three-way interaction between CP, HAZ and HAZ-L (*F*(1,164) = 4.29, *P* = 0.04, *η*_p_^2^ = 0.03, CI = −5.47 to −0.13). As shown in Fig. 7a, infants of normal height with higher linear growth had better outcomes in year 2. By contrast, infants of normal height with lower linear growth fared poorer, except for infants with higher CP scores in year 1. These infants with higher CP scores showed good problem-solving scores in year 2. This finding suggests that VWM abilities in infancy might be protective against cognitive deficits associated with poor linear growth. In general, stunted infants showed poorer problem-solving scores in year 2.

Next, we asked whether the load-dependent patterns of activation observed in laIPS (Fig. 4a) predicted cognitive outcomes 1 year later. We ran separate models for HbO and HbR concentrations. Even though normal height infants elicited greatest activation at the low load, we did not observe any associations with the problem-solving score from this condition. However, as shown in Fig. 7b, there was a significant interaction between HbR concentration in laIPS and HAZ scores (*t*(167) = −2.46, *P* = 0.015, *η*_p_^2^ = 0.03, CI = −8.72 to −0.42). In normal height infants, greater laIPS activation (that is, negative HbR concentration) was associated with better problem-solving scores in year 2. Follow-up tests showed no robust associations between brain activation and problem-solving scores for stunted infants (*P* > 0.05).

## Discussion

Stunting in the first 1,000 days is associated with poor cognitive, academic and economic outcomes in later life, yet the neurocognitive mechanisms underlying these relationships early in development are unknown. We proposed that stunting in infancy might impact early emerging systems such as VWM, a critical system that is predictive of individual differences in cognitive function and academic outcomes. Toward this end, we examined how stunting impacts VWM performance and brain function in the first year in a large sample of infants from rural India; we also examined cognitive outcomes one year later.

Consistent with findings from Western settings, behavioural performance in the VWM task declined with increasing load^16,17^. Further, infants in this study engaged key hubs in the attention and VWM networks^30^ such as rIFG, laIPS and rTPJ as a function of VWM load and task-related performance. We also replicated a critical finding from our previous work in this population: infants who sustained longer looking to the changing side (greater CP scores) showed suppression in rIFG^18^. The inferior frontal junction is purported to act as a switching hub between the dorsal and ventral attention networks, with suppression implying less frequent switching between top-down goal-driven attention and bottom-up reorienting of attention to salient/distracting stimuli^31^. We suggest that infants who sustained longer looking towards the changing side and showed better VWM performance, infrequently reoriented attention to the unchanging side via IFG suppression. Increased frontal activation has also been linked to processing and storage of irrelevant distractor information in visual and spatial working memory tasks in children^32^. Thus, in the current study, greater rIFG activation in infants with poorer CP scores might also reflect processing of information from the distracting, unchanging side.

Previous studies with children and adults have shown that active working memory maintenance is associated with bilateral posterior parietal activation^21,22,24,33^. Further, parietal lateralization has been linked to the type of working memory used, with verbal working memory activating left parietal cortex and some studies showing greater involvement of right parietal cortex in visuospatial working memory^34^. In the current study, only left aIPS was engaged across multiple effects of age, load, chromophore, CP score and HAZ in infants, suggesting a key role for this brain region in VWM processing. This is consistent with evidence from an urban US sample which revealed robust relationships between performance in the preferential-looking VWM task and activation in the left hemisphere in infancy^17^. Environmental/contextual differences might explain the emergence of lateralized activation in the first year of life. For example, bilingual language, compared to monolingual language exposure in infants has been associated with bilateral recruitment of the frontal cortex during a non-linguistic attentional orienting task^35^.

We also replicated another key effect from our previous study of urban US infants: infants of normal height showed a load-dependent modulation in the laIPS with high activation at the low load and lower activation at higher loads. This pattern is consistent with similar effects in 3- and 4-year-old children^36^ as well as aging adults^37^. Interestingly, this pattern contrasts with findings from adults; increasing VWM load is associated with increasing aIPS activation until a capacity limit is met; this is followed by an asymptote in brain activation^23,33,38^. Collectively, these findings suggest that at high loads, infants, children and aging adults fail to maintain a near-capacity number of items (and an asymptotic level of brain activity). How, then, were some infants able to show above-chance levels of responding at the medium and high loads? On some of these trials, infants start visual exploration on the changing side. Detection of novelty in this case does not require robust working memory ability as there is a change after each flash. Thus, it is possible that some infants show above-chance responding because they started on the changing side, detected novelty and this novelty sustains looking to this display^39^.

We observed a refinement of laIPS activation with age and enhanced VWM performance. Specifically, 6-month-olds and low-performing 9-month-olds showed a greater extent of laIPS engagement. Moreover, in children of normal height (compared to stunted infants), both age groups showed greater laIPS activation in lower-performing infants. These findings are new and suggest a refinement of laIPS activation between 6 and 9 months in infants from rural India consistent with behavioural evidence of a change in VWM capacity between these ages in urban infants^17^. The extent of spatial refinement of laIPS activation underscores the precision of the image reconstruction techniques we used (for a comparison to a channel-based fNIRS approach; Supplementary Information).

The present study also revealed how stunting impacts looking behaviour. We found that stunted infants showed a poorer ability to detect and stay fixated on the changing side. The linear association between stunting status and CP scores was stronger in 6-month-old infants compared to 9-month-old infants suggesting that stunting-related impact on VWM processing might be mitigated with age. However, our brain imaging results imply otherwise. Stunting modulated activation in laIPS, rTPJ and lDLPFC—key regions in the canonical frontotemporoparietal VWM network.

Unlike infants of normal height, stunted infants more consistently showed weak activation in the laIPS—only high-performing 6-month-olds showed robust engagement of this brain region. In the absence of active laIPS engagement, how did some stunted 9-month-olds achieve above-chance performance? Our findings suggest that these infants recruited the lDLPFC, a region involved in the top-down modulation of processing in posterior parietal cortex. Thus, while 9-month-old infants of normal height engaged laIPS to achieve sustained looking to the changing side, stunted 9-month-old infants might have engaged the frontal cortex to compensate for weak activation in laIPS. Notably, this pattern of dependence on frontal cortex activation was also observed in younger infants of normal height. Taken together, our findings suggest that stunting status might be associated with impairments or delays in the functional activation of the VWM network.

Stunting status also selectively modulated rTPJ function. The rTPJ is thought to act as a ‘circuit-breaker’ with greater activation associated with bottom-up reorienting of attention away from ongoing task-related processes and towards salient and/or irrelevant stimuli^21,22,40,41^. In VWM processing in adults, increasing VWM load is associated with increased laIPS activation and rTPJ suppression^21^. In agreement with this evidence, we found that infants of normal height who showed better VWM performance and less distractibility engaged laIPS to successfully maintain representations of the items and supressed rTPJ to prevent frequent reorientation of attention. On the other hand, in stunted infants, rTPJ suppression was associated with poor CP scores suggesting that some of these infants might have become ‘stuck’ on the unchanging side.

As a final question, we asked whether these neurocognitive patterns impacted cognitive outcomes 1 year later. Our findings suggest that VWM function might act as a protective factor against deficits in more complex cognitive functions in later years, consistent with evidence showing that VWM function in infancy is a reliable predictor of cognitive outcomes 11 years later^42^. Concretely, at-risk infants with low height-for-age scores in infancy and lower linear growth from the first to the second year of life showed better problem-solving scores in year 2 if they had good VWM performance in year 1. We then asked whether laIPS activation in infancy was linked to better cognitive outcomes in year 2. Interestingly, infants of normal height with greater laIPS activation in year 1 demonstrated higher cognitive scores in year 2. It is important to contextualize these findings given that multiple factors may promote healthy VWM processing in the first year of life (see ref. ^43^ for a review of biophysiological pathways impacted by poverty). Infants of normal height might be reared in higher-resource homes with access to more cognitive materials and activities compared to stunted infants. Moreover, exposure to less stressful environments may promote opportunities for rich quantity and quality of explorative play leading to typical, healthy development of attention and VWM networks. Previous work from our group showed that parent-reported frequency of stressful life events was predictive of left parietal cortex engagement during a VWM task in low-performing preschool children^33^. Stunted children might also be exposed to living conditions with poor sanitization, poor hygiene, pathogen exposure and environmental contaminants leading to chronic infections and malnutrition. Resulting general malaise or sickness could lead to diminished opportunities for explorative play and social learning impacting cognitive development in the first year of life. All these conditions could also lead to anatomical differences in brain structure and connectivity as well as reduced cortical activity, eventually affecting VWM and attention processing pathways.

Our findings are consistent with studies suggesting that stunted children show longer-term working memory deficits. For example, stunting in the first 2 years of life is associated with poor performance in visual and spatial working memory function in 17- to 18-year-old adolescents in Jamaica^3^. Similarly, another study found that malnourished children across the age groups of 5–7 years and 8–10 years in India showed poorer performance on a working memory task compared to adequately nourished children in these age groups^44^.

Future work should address some methodological limitations in the current study. First, we used context-matched templates for infants for whom we did not have anatomical scans. Follow-up research should quantitatively examine the impact of using templates with infants from low-resource settings. Second, future work should evaluate how motion detection and motion correction parameters should be chosen for studies with at-risk infants, such that findings are representative of individual as well as group-level differences.

To summarize, the current study makes a unique contribution to this body of work by using neuroimaging tools to study the most at-risk infants globally, demonstrating that such tools can be successfully deployed to investigate and identify neurocognitive mechanisms in rural settings^45,46^. We show that stunting impacts looking behaviour and is associated with modulation of neural activity in key hubs of the VWM and attention brain networks and, further, that these neurocognitive patterns are associated with later cognitive outcomes. Our findings also paint a picture of hope in that better VWM function in infancy may confer some neurocognitive protection, at least for short-for-age infants. Given that previous work suggests that visual cognition can be enhanced via caregiver-based interventions^47^, this could provide an avenue for future efforts to boost VWM function in infancy before stunting-related cognitive deficits take hold.

## Methods

The research complies with all relevant ethical regulations. Approval for this study was provided by the Institutional Ethics Committee at the Community Empowerment Lab (CELIEC/2017002), Lucknow, India.

### Participants

Families with infants aged 6 months ± 15 days or 9 months ± 15 days from the villages in and around Shivgarh in the district of Raebarelli, Uttar Pradesh, India were contacted by researchers from the Community Empowerment Lab (CEL). Infants born to parents screened with colour vision deficits (due to the nature of the VWM task) or any congenital problems or gestational age <26 weeks at birth were excluded from the study. Infants were enroled across four waves of data collection separated by 3 months from May 2017 to February 2018 (year 1). Approximately 30 6-month-olds and 30 9-month-olds were enroled in each wave. Infants were also followed up for another year from 2018 to 2019 (year 2).

Across both years, each family participated in laboratory visits, home visits and magnetic resonance imaging (MRI) visits. During the laboratory visit, physical growth measurements, behavioural and brain imaging (fNIRS) data and cognitive assessment data were collected. During the home visit, physical growth measurements were taken. During the MRI visit, physical growth measurements and anatomical scans were collected. If it was not possible to collect all the data in a single visit, the family was invited for multiple visits scheduled in close succession. The timelines of these visits were as follows: (1) a laboratory visit in 6 months (year 1) and 18 months (year 2) for the 6-month-old cohort and at 9 months (year 1) and 21 months (year 2) for the 9-month-old cohort, (2) a home visit every 3 months thereafter in year 1 and year 2 (for example, at 9, 12, 15, 21, 24 and 27 months for the 6-month-old cohort and at 12, 15, 18, 24, 27 and 30 months for the 9-month-old cohort) and (3) an MRI visit in year 1 (for example, at 6 months for the 6-month-old cohort and 9 months for the 9-month-old cohort). Note that the assessments reported here were a subset of the full research protocol for the project. The full list of assessments can be found in the Supplementary Information.

The 277 families met the inclusion criteria and gave due consent. The study had no provision for compensation; however, the participant families were offered a gift hamper with baby items as a token of appreciation. From this sample, 37 children did not complete the first in-take assessment (19 6-month-olds and 17 9-month-olds). The remaining 240 families were enroled into the study. Data from 17 infants were excluded from all analyses due to problems with the behavioural and neuroimaging data collection and processing (not enough behavioural data in 9 infants, technical problems with the neuroimaging system for 7 infants and neuroimaging data lost due to motion artifacts from 1 infant). We included infants in the analyses if they had usable fNIRS for at least one load of the VWM task. Data from 223 infants were included in the final analyses (see Supplementary Table 1 for demographic details on this sample). Some families also provided consent to use their images for knowledge and research purposes.

#### Materials and procedure

##### General procedures for the laboratory visit

Families were transported in groups from their homes to the CEL Facility in Shivgarh. Three or four researchers were present during the sessions. The family were first escorted to the waiting room of the facility. Some groups of families were also provided a tour of the facility and a demonstration of the procedures to make them feel more comfortable and allow them to ask questions. Next, the families were escorted back to the waiting room where informed consent was sought. Participants’ caregivers provided written informed consent; where caregivers were illiterate, a witness gave signed consent accompanied by a thumb impression of the caregiver in place of a signature. After consent was obtained, physical measurements of the infant were taken. The infant’s head circumference was also measured to prepare an appropriately sized cap for fNIRS data collection.

Next, the parent and the infant were escorted to the fNIRS assessment room. The room was coloured in a neutral grey to prevent distraction of the infant. The mother was seated on a chair and the infant was placed on the mother’s lap ~100 cm away from the TV screen (Fig. 1b). A cartoon was played on a television screen to engage the infant. When the infant looked comfortable, two researchers placed an appropriately sized fNIRS cap on the head and fastened the chinstrap to hold it in place. After adjusting fNIRS signals (for example, clearing hair from under individual fNIRS optodes), one researcher proceeded to use a Polhemus sensor to collect coordinates of the scalp landmarks and source and detector positions, while a second researcher placed a calibration sticker on the infant’s forehead and set up the eye-tracker to record eye-movements from the infant. A five-point calibration sequence was played on the monitor to ensure correct eye-tracking at the top, bottom, left, right and central parts of the screen.

After this, the VWM task was presented and video recordings, eye-tracking and fNIRS data collection ensued. If the infant showed signs of distress, cartoon clips were played in between trials. Breaks were provided if the infant needed to be fed, fell asleep or could not be calmed even after the use of the cartoon clips. The family was escorted back to the waiting room after the completion of the assessment or if the infant and/or mother needed a break from the assessment. In year 2, the mother and infant were escorted to another room to administer the ASQ assessment. At the end of each laboratory session, families received a small gift for participating in the study.

##### Physical growth measurements

Physical growth measurements were taken during laboratory visits, home visits and MRI visits unless two or more visits were close in time, in which case, a single measurement was used for that time point. Measurements of head circumference, mid-upper arm circumference, calf circumference, infant’s weight and infant’s length were taken by two members of the research team who were trained through a standardized workshop. Head circumference, mid-upper arm circumference and calf circumference were measured twice using a seca measurement tape. Measurements were repeated if there were discrepancies of >7 mm between two measurements. An infantometer was used for measuring the child’s length from head to heel with 1 mm precision. A digital seca weighing scale was used to measure the baby’s weight with 10 g precision.

##### VWM task

Infants were presented with a preferential-looking VWM task^16^ during the laboratory visit. A PC running Experiment Builder (SR Research) was used to present the task on a 42-inch LCD television screen. Infants sat on their parents’ lap ~100 cm away from the screen. Each stimulus display area was 29.5 cm in width and 21 cm in height, with a 21 cm gap between the display on the left and right (each coloured square was about 5 × 5 cm^2^). The displays had a solid grey background. The colours of the squares presented on each display were selected from a set of nine colours: green (RGB: 0, 153, 0), brown (128, 64, 32), black (0, 0, 0), violet (128, 0, 128), cyan (128, 255, 255), yellow (255, 255, 0), blue (0, 0, 255), white (255, 255, 255) and red (255, 0, 0). On a display, the colours of the squares differed from each other but colours could be repeated between the displays (that is, the same colour could appear on both displays). The positions of the squares on each display were randomly selected from a 3 × 3 grid of possible positions. Eye-movement data were recorded using an Eyelink 1000 Plus eye-tracker (SR Research) operating in binocular mode with a sampling rate of 500 Hz. Additionally, one camera recorded a view of the infants’ face and another camera recorded the television display. These video recordings were used to extract looking data when eye-tracking information was not available (due to technical problems, reflectance, poor lighting and loss of calibration).

Each trial started with a dynamic attention cue. Once the eye-tracker/experimenter detected that the infant was looking at the attention-getter, the task proceeded to the VWM displays. Each trial consisted of side-by-side displays of coloured squares that appeared for 500 ms and disappeared for 250 ms for a trial duration of 10 s. Each trial was followed by a minimal intertrial interval of 5 s, however, this period was typically longer as the trial was not initiated until the infant looked at the display following the dynamic attention cue. On the ‘unchanging’ side, the colours of the squares remained the same across each flash, while on the ‘changing’ side, one square changed its colour across each flash. VWM load was manipulated by varying the number of squares on each side across trials (one, two or three squares on each side). The aim was to present each infant with 36 total trials in six blocks of six trials, although where the infant and parent were willing to continue, additional blocks were sometimes collected. Each block contained two trials for each load, one with the changing side on the left, one with the changing side on the right. Order of trials was randomized in each block. Where necessary, participants could take a break between blocks.

##### Functional near-infrared spectroscopy data acquisition

The fNIRS data were collected from infants as they engaged with the VWM task during the laboratory visit. A TechEn CW7 system and software (12 sources and 24 detectors) with wavelengths of 830 and 690 nm and sampling rate of 25 Hz was used to collect brain function data. Fibre optic cables were used to carry light from the TechEn system to a cap with a customized probe geometry of 36 channels overlying the frontal, parietal and temporal cortices (Fig. 1c). A laptop connected to the fNIRS system recorded and displayed data as it was being collected. This laptop was also connected to the Experiment Builder computer to synchronize fNIRS data with the start of each trial of the task. A Polhemus Patriot Motion Sensor was used to digitize scalp landmarks and positions of sources and detectors on the cap.

##### MRI data acquisition

Anatomical data were collected on a Philips Achieva 3T MRI scanner equipped with 12-channel head RF array in an MRI Facility in Lucknow, India. The protocol used volumetric T_1_-weighted SPGR. All imaging was performed during natural sleep^48^. Acquisition parameters were as follows: for T_1_ SPGR: field of view = 19 × 19 cm^2^; slice thickness = 1.2 mm; acquisition matrix = 94 × 194; flip angle = 9°; echo time = 3.72 ms; repetition time = 9.5 ms; and receiver bandwidth = 270 Hz per voxel.

##### Ages-and-stages questionnaire III assessment

The ASQ was administered during laboratory visits in year 2 when the infants were 18 months (for the 6-month-old cohort) or 21 months (for the 9-month-old cohort). The appropriate ASQ questionnaire for each infant was selected using the online ASQ calculator (https://agesandstages.com/free-resources/asq-calculator/). While ASQ is designed as a screening questionnaire to be completed by parents, we adapted its administration to improve the reliability of the data. Specifically, a trained assessor administered the ASQ in collaboration with the parent. In cases where questions from the ASQ materials kit asked about behaviours that could be elicited in the laboratory (for example, ‘When you ask your child to, does he go into another room to find a familiar toy or object?’), these tasks were completed ‘live’, ensuring that the child was given ample time to perform each task. In the event that the child was unable to perform the task or the question was not amenable to live assessment, the mother’s verbal report on the question was taken as the response. The ASQ yields five subscales of development: communication, gross motor, fine motor, problem-solving and personal-social. Each subscale contained six questions, making up a total of 30 questions on the form. For this study, we focused on the problem-solving scale as it was most directly related to VWM function and we were interested in investigating later cognitive outcomes.

#### Methods of analysis

All statistical tests included in the manuscript and supplementary analyses used two-tailed statistical tests.

##### Physical growth measurement analyses

A height-for-age *z*-score was calculated by dividing the difference between each infant’s height and the age-specific height obtained from World Health Organization (WHO) growth standards by the age-specific standard deviation. Participants contributed between 1 and 11 observations for their height-for-age *z*-score across the study. On average, participants contributed 6.54 observations (s.d. = 1.92 observations), 83 days apart from each other (s.d*.* = 24.5 days). To obtain individual estimates of physical growth, a linear mixed effects model was run, taking height-for-age *z*-scores as the dependent variable and participant age in days as an independent variable. As the trend was not perfectly linear, a quadratic transformation of age in days was also added to the model. Both age and age squared were orthogonal, that is, they were independently scaled and centred to avoid autocorrelation. The models had a random effect structure such that the intercept, the linear age term and the quadratic age term were nested by participant. The random effect coefficients of the model were then extracted for each participant, such that each participant had an intercept term (HAZ), a linear age term (HAZ-L) and a quadratic term. As age in days was scaled and centred, the intercept term, used in most analyses, represents an area under the curve, rather than an initial estimate. HAZ scores were available for all 223 infants.

Measurements were also taken for weight-adjusted *z*-score, weight for length, arm circumference and head circumference as well as the participants’ body mass index. All of these, excluding body mass index, were modelled in an identical fashion to the HAZ scores to extract individual coefficients. While all of these measurements share a certain amount of variance, HAZ was selected as the variable of interest as it had the highest correlation with socioeconomic status (*t*(238) = 5.912, *P* < 0.001, Pearson correlation = 0.358).

##### VWM task analyses

On average, infants completed 21.8 trials (s.d. = 10.2). SR Research Data Viewer was used to export frame-by-frame eye-tracking data. The areas of interest around the two objects on the screen was modified such that the eye-tracking data would match video-coded data where the primary categories were ‘left’, ‘right’ and ‘away’. Manually coded data based on video recordings were used to replace trials where no eye-tracking information was available. Datavyu (https://datavyu.org/) was used to manually code these video recordings capturing the television screen and the infant’s face. A neutral observer coded the infant’s eye-movements into ‘left’, ‘right and ‘away’ looks for each frame for each trial. We computed reliability using Cohen’s Kappa, a statistic that looks at percentage agreement across categories normed by the base rate of each category. Kappa values from 0.6 to 0.8 indicate substantial reliability. Scores >0.8 indicate almost perfect agreement. Overall, we coded 15% of the data to check reliabilities. Reliabilities were very good with a mean Kappa of 0.73 for the 6-month-old cohort and a mean Kappa of 0.83 for the 9-month-old cohort. Once coded, data were then exported in a format compatible with the eye-tracking data. The manually coded trials made up 31.9% of the total number of trials.

Data preprocessing was carried out using the R package eyetrackingR^49^. Two key measures were obtained from the data—TLT and CP. TLT for each trial was calculated as the sum of time spent looking at both displays. CP was calculated as the time spent looking at the changing side divided by the total looking to both displays during a critical time window of 1,500–6,500 ms for each trial (out of the 10,000 ms trial window). The first 1,500 ms comprised of the first two flickers (2 × (250 ms off and 500 ms on period)) was excluded to allow infants to explore the displays and, potentially, detect the changing and unchanging sides. The final 3,500 ms was also excluded because the number of eye-tracking samples diminished as attention waned. Trials during which 75% of the data were coded as not looking at the displays were excluded from further analyses. Following these processing steps, out of the 223 children included in the analyses, 214 children contributed CP scores for load 1, 209 children contributed CP scores for load 2 and 214 children contributed CP scores for load 3.

A linear model with the CP score as the dependent variable and the age (6 or 9 months), load (1, 2 or 3), HAZ score and TLT (in milliseconds) as independent variables was used to model the behavioural data. All interactions between independent variables were included. Participant socioeconomic status (measured using the Kuppuswamy scale^50^) and other related variables (for example, nutrition information) were added in individually to assess whether there was an improvement to the model fit; however, these additional predictors were discarded as they either were colinear with HAZ or did not contribute substantially to model fit. An attempt was made to allow for the individual-level variance by adding a random intercept for each participant but this resulted in a singular fit, indicating the random effect was estimated at approximately zero.

##### fNIRS data preprocessing

For the fNIRS analyses, 221 children had fNIRS data for load 1 (out of the 223 infants), 220 children had fNIRS data for load 2 and 221 children had fNIRS data for load 3. The fNIRS data were preprocessed using EasyNIRS in HOMER2 (ref. ^51^). Raw data were pruned using the enPruneChannels function (dRange = 1 × 10^4^ to 1 × 10^7^, SNRthresh = 1, SDrange = 0–45). An average of 29% of the channels across runs and infants were pruned/lost/rejected. Next, the hmrIntensity2OD function was used to convert data to optical density units. Motion artifacts were identified and corrected using targeted principal components analysis through the hmrMotionCorrectPCArecurse function (tMotion = 1.0, tMask = 1.0, StdevThresh = 50 and AmpThresh = 0.5, nSV = 0.97, maxIter=5). The corrected data were examined for uncorrected motion artifacts using the hmrMotionArtifactByChannel function (tMotion = 1.0, tMask = 1.0, StdevThresh = 50 and AmpThresh = 0.5). If these artifacts fell within −1 to 18 s of a trial, the associated trial onset trigger was removed from further data processing using the enStimRejection function. These criteria were set on the basis of previous work examining motion-processing algorithms in a development dataset^51^. An average of 6% of the trials across runs and infants were lost due to motion artifacts. These data were then band-pass filtered using the hmrBandpassFilt function with high-pass and low-pass cut-off frequencies of 0.016 and 0.5 Hz, respectively. The processed data were further analysed using Image Reconstruction techniques described below.

Note that these motion correction parameters were based on data from a VWM study with 3.5- to 4.5-year-old children. More recent analyses of infant fNIRS data^52,53^ have recommended similar motion correction parameters with one notable difference: these studies suggest using StdevThresh = 15. This difference is not surprising as setting this parameter is time consuming and subjective^53^. To examine how this would impact our findings, we re-ran all analyses with StdevThresh = 15. Although findings from this re-analysis were consistent with our main results (Supplementary Information), the lower value yielded very high data loss (mean data loss with StdevThresh of 50 = 6.19%; mean data loss with StdevThresh of 15 = 37.22%), particularly for stunted infants (mean data loss with StdevThresh of 15 = 41.2%). This is a very high percentage of data loss, raising questions about whether findings obtained from using this conservative threshold are representative of individual and/or group-level estimates of brain function in at-risk infants. Given this, we discuss findings from using StdevThresh = 50 in the final analyses.

##### Creating head models for fNIRS analyses

To create a head model for each infant, we used the anatomical MRI scan if it was available. Out of the 223 children included in the analyses, anatomical T_1_-weighted images were available for 72 6-month-old infants and 70 9-month-old infants. The remaining infants did not have an anatomical scan (45 6-month-olds and 36 9-month-olds). If a scan was not available, we used an age-specific MRI template. A 6-month-old template and a 9-month-old template were created from the available scans of 15 boys and 15 girls for each specific age, using a multistep registration procedure^54^. This procedure was carried out using antsMultivariateTemplateConstruction2 provided by ANTS 2.1. Briefly, all the images were linearly aligned and averaged to provide a template estimate. Then, all images were nonlinearly aligned to this initial estimate. The results were averaged to provide an improved estimate. This process was repeated ten times to construct the final estimate. The 6-month-old template was used for the 45 6-month-olds who did not have anatomical scans and the 9-month-old template was used for the 36 9-month-old infants who did not have anatomical scans.

Next, head models were created from the anatomical scans and age-specific templates using tools available in AFNI v.2 (https://afni.nimh.nih.gov). We describe this briefly below. First, images were rotated using the 3dRotate such that the nose was rotated towards the *y* axis of the MRI scanner. The images were then resampled into a standard right-axial-superior orientation using 3dResample. If large variations in signal quality were present in the image, 3dUnifize was used to do bias field correction. The image was put into anterior commissure (AC)–posterior commissure (PC) alignment. To do this, a brain mask was created and the brain was extracted from the image and aligned using the auto_tlrc function. The rigid body transform from the resulting transform was then used to reorient the image and brain mask into AC–PC alignment. The image was segmented into grey matter, white matter and cerebrospinal fluid using 3dSeg. A corresponding model representing the outer surface of the scalp was generated by estimating the optimal threshold between the background air and the foreground head using 3dClipLevel. This threshold was then applied to the image and holes in the resulting mask were filled using 3dinfill. This segmentation representing the head was combined with the brain segmentation to generate a label map that contained four labels: grey matter, white matter, cerebrospinal fluid and a combination of skull and scalp. These head volume images were used for fNIRS Image reconstruction analyses described below.

##### Generating forward models and fNIRS image reconstruction

Details of the methodological pipeline used for image reconstruction are presented elsewhere^55^. We outline the steps below. First, we corrected for variations in scalp landmarks and positions of sources and detectors during digitization (for example, infant movement) using a three-step method in the digitizeR package. This method sequentially compares and aligns user-specified Euclidean distances between sources and detectors for an individual probe geometry with available templates for specific cap sizes. Head volumes created from segmenting the T_1_-weighted anatomical scans or age-specific templates were imported into AtlasViewerGUI in HOMER2 in Matlab v.2016b (refs. ^30,56^). Each infant’s digitized scalp landmarks and probe geometry was projected onto each head volume. Monte Carlo simulations were run with 100 million photons to generate sensitivity profiles for each channel and wavelength. The head volume and sensitivity profiles were converted to NIFTII images. Next, image reconstruction techniques using NeuroDOT tools^55,57^ were used to integrate the head volume and sensitivity profiles with the processed fNIRS data to obtain voxel-wise relative changes in oxygenated (HbO) and de-oxygenated (HbR) concentrations.

A general linear model with three regressors (loads 1, 2, 3) was separately run for each chromophore and infant. We used a haemodynamic response function derived from DOT data for HbO and HbR data^55^. Each trial was modelled with a 10 s boxcar and variable intertrial intervals. The intertrial interval was a minimum of 5 s but could be typically longer as the trial was not initiated until the infant looked at the display following the dynamic attention cue. To control for the variability in the number of trials per condition and infant, we computed a weighted average of beta coefficient images per load, chromophore and infant.

##### Registration to overall study template

The image-based fNIRS beta coefficient images from all infants were aligned to the same space by using an overall study template. This overall study template was created by repeating the same process described in the multistep registration procedure^54^, using each age-specific template (6-, 9-, 12-, 15-, 18-, 24- and 30-month-old)^58^—see section on Creating head models for fNIRS analyses above. For the current study, we used this overall study template, instead of a template generated from combining only the 6- and 9-month-old templates, to provide consistency across current and future investigations on this project.

##### Group analyses

For group analyses, only voxels that contained data from 70% of the infants were included. To achieve this, the beta coefficient map for each condition, chromophore and infant was masked and summed together to create one image. Only those voxels that contained at least 70% data were extracted to create the group mask. This group mask was used in the model below. For group analyses, the coregistered beta coefficient maps were entered into a linear mixed effects model using 3dLME in AFNI with load (1, 2, 3), chromophore (HbO and HbR) and age (6- and 9-month-olds) as within-subject factors and CP and HAZ as quantitative predictors. For children who did not have a CP score at a load, an average CP score was calculated for that load from age-matched, gender-matched and socioeconomic status-matched infants. An average CP score was used for 9 infants for load 1, 14 infants for load 2 and 9 infants for load 3. After running the linear mixed effects model, 3dFWHM was used to estimate the empirical autocorrelation function in our data and fit a mixed autocorrelation function model to this function. 3dClustSim was run on the group brain mask with a voxel-wise *P* threshold of 0.01, alpha threshold of 0.05, 10,000 iterations, one-sided thresholding, nearest-neighbour = 1 and with a minimum cluster size of 278 voxels. 3dClusterize was used to threshold the main effect and interaction effect images. Average beta values were extracted from the thresholded clusters using 3dROIstats in AFNI. Labels for significant clusters of activation were created based on regions of interest (ROIs) from VWM fMRI studies^30^. These ROIs in MNI space were coregistered to align with the overall study template. To assign a label to each significant cluster, Euclidean distances were calculated between the centre of mass of each ROI and each significant cluster. The ROI with the minimum distance to a cluster was used as the label. The range of distances between each cluster and final assigned label was 7.5–22.7 mm.

##### ASQ assessment analyses

Out of 223 children included in the analyses, 172 children had scores for the ASQ assessment. The rest of the families did not complete the assessment due to illness, non-attendance or other factors. Non-standardized scores from the ASQ were used as there are no standardized norms for children from rural India. To examine whether behavioural performance in year 1 was related to the problem-solving score in year 2, we ran a linear model with the problem-solving score as the dependent variable and CP scores, HAZ scores and HAZ-L as predictors. We included HAZ-L because change in physical growth over time was expected to be an important contributor to problem-solving score measured in year 2.

To examine whether brain function in year 1 was related to cognitive outcomes in year 2, we ran six linear models—one per chromophore (HbO and HbR) and load (1, 2, 3) for the laIPS cluster from the interaction between HAZ, load and chromophore. In each model, the dependent variable was problem-solving score and the independent variable was laIPS activation. We also included the HAZ score as a predictor since laIPS activation was associated with HAZ. Note that we tested whether HAZ-L contributed significantly to these models; this was not the case, so this term was excluded from the final models. All models were checked with a Q–Q plot of the residuals and using the DHARMa^59^ package v.0.4.3 in R. As there was some level of dispersion in the data, models were run using robust regression (using the robustbase package in R) and model outliers were also checked using Cook’s distance, indicating no problematic outliers. For these models, effect size was calculated using linear regression with the same variables.

### Reporting summary

Further information on research design is available in the Nature Portfolio Reporting Summary linked to this article.

### Supplementary information

Supplementary Information Supplementary Tables 1–3, Figs. 1–6 and Discussion.

Reporting Summary

## Data Availability

All behavioural and brain data used in statistical analyses, including scripts and code, are publicly available on 10.17605/OSF.IO/KC3N8.

## Code availability

Code used for statistical analyses is publicly available on 10.17605/OSF.IO/KC3N8. fNIRS analysis pipeline is publicly available at https://github.com/developmentaldynamicslab/MRI-NIRS_Pipeline.
