# Supplementary material for: Stunting in infancy is associated with atypical activation of working memory and attention networks
Source: Nat Hum Behav. 2023 Oct 26;7(12):2199–211. doi: 10.1038/s41562-023-01725-3 (PMC10730391; doi:10.1038/s41562-023-01725-3)
Supplement: Supplementary file 1 — Supplementary Tables 1–3, Figs. 1–6 and Discussion. [file 41562_2023_1725_MOESM1_ESM.pdf]

nature human behaviour

Article

https://doi.org/10.1038/s41562-023-01725-3

Stunting in infancy is associated with atypical activation of working memory and attention networks

In the format provided by the authors and unedited

Table of Contents

1. Demographic and socioeconomic distribution of information for the sample (Supplementary table 1).
2. Three-way interaction between load, TLT and HAZ (Supplementary figure 1).
3. Three-way interaction between load, CP score and chromophore in right frontal eye fields (Supplementary figure 2).
4. Assessments conducted in Project INDIA.
5. Supplementary analyses of fNIRS data using channel-based approach.
6. Supplementary analysis of fNIRS data with StdevThresh of 15.

				Mean	Standard deviation	Minimum	Maximum
**Sample size**	6-month-olds	High SES	Male	32			
			Female	26			
		Low SES	Male	28			
			Female	31			
	9-month-olds	High SES	Male	23			
			Female	25			
		Low SES	Male	30			
			Female	28			
**Age (days)**	6-month-olds	High SES	Male	176.8	15.6	131	207
			Female	184.5	12.4	157	209
		Low SES	Male	181	14.7	144	212
			Female	183.7	14.9	150	220
	9-month-olds	High SES	Male	270	13.8	226	289
			Female	266.3	14.6	238	288
		Low SES	Male	266.4	13	240	289
			Female	266	13.8	230	288
**Gestational age (weeks)**	6-month-olds	High SES	Male	38.4	1.9	34	42
			Female	38.1	2.8	30	40
		Low SES	Male	36.3	3.6	28	41
			Female	36.6	4.4	26	42
	9-month-olds	High SES	Male	38.1	2.6	32	42
			Female	38.3	2.6	33	42
		Low SES	Male	38.5	2.5	32	42
			Female	36.1	3.7	27	42
**HAZ score**	6-month-olds	High SES	Male	-1.2	0.9	-2.9	1.2
			Female	-1.7	0.8	-3.1	0.9
		Low SES	Male	-2.1	0.8	-3.5	-0.1
			Female	-1.9	0.9	-3.7	-0.1
	9-month-olds	High SES	Male	-1.6	1.1	-3.4	1.2
			Female	-1.4	0.9	-2.9	0.8
		Low SES	Male	-1.8	0.8	-3.3	-0.3
			Female	-2	0.9	-4	0
**Head circumference (cm)**	6-month-olds	High SES	Male	42.4	1.3	40	46
			Female	41.2	1.3	38	43.5
		Low SES	Male	42.2	1.5	39	45
			Female	41.2	1.2	39	43.5
	9-month-olds	High SES	Male	43.6	1.4	39.5	46
			Female	42.8	1.4	40	46
		Low SES	Male	44	1.5	40.5	46

			Female	42.9	1.2	41	46.5
**Mother Age (years)**	6-month-olds	High SES	Male	25.5	4.3	19	40
			Female	25	3.8	20	36
		Low SES	Male	28.7	5.3	21	47
			Female	29	4.8	20	40
	9-month-olds	High SES	Male	23.8	2.9	18	30
			Female	24.5	2.4	20	30
		Low SES	Male	27.4	5.3	20	42
			Female	26.6	5.3	18	40
**Father Age (years)**	6-month-olds	High SES	Male	28.1	4.7	21	45
			Female	27.6	4.3	21	38
		Low SES	Male	31.2	5.3	23	45
			Female	31.7	6.2	22	50
	9-month-olds	High SES	Male	26.6	3	21	33
			Female	27.2	2.7	21	33
		Low SES	Male	29.8	6	23	47
			Female	29.8	5.9	22	45
**Mother Education (years)**	6-month-olds	High SES	Male	4.8	0.9	4	6
			Female	4.5	0.7	4	6
		Low SES	Male	1.5	0.5	1	2
			Female	1.4	0.5	1	2
	9-month-olds	High SES	Male	4.7	0.9	4	6
			Female	4.8	0.8	4	6
		Low SES	Male	1.3	0.5	1	2
			Female	1.5	0.5	1	2
**Father Education (years)**	6-month-olds	High SES	Male	4.7	0.8	4	6
			Female	4.7	0.8	4	6
		Low SES	Male	1.6	0.5	1	2
			Female	1.9	0.3	1	2
	9-month-olds	High SES	Male	4.4	0.7	4	6
			Female	5	0.8	4	6
		Low SES	Male	1.6	0.5	1	2
			Female	1.7	0.5	1	2
**Income (INR)**	6-month-olds	High SES	Male	98637.4	86036	15000	480000
			Female	89807.7	61356.9	12000	225000
		Low SES	Male	43321.4	21813.7	12000	100000
			Female	48163	32695.4	10000	120000
	9-month-olds	High SES	Male	92434.8	79230.5	20000	350000
			Female	119440	95327.4	10000	450000
		Low SES	Male	56666.7	35240.9	8000	150000
			Female	45903.6	25165.9	8000	96000
**SES score**			Male	13	4.1	7	26

	6-month-olds	High SES	Female	13	4.1	8	22
		Low SES	Male	6.6	1.7	4	9
			Female	7.3	2.2	4	13
	9-month-olds	High SES	Male	11.1	3.1	7	20
			Female	13.3	3.3	8	20
		Low SES	Male	7.4	2.7	4	16
			Female	7	2.7	4	16

Supplementary Table 1. Demographic and socioeconomic distribution for each group. Note that SES categorization (low vs high) was based on an initial assessment of parental education at enrolment, while ‘SES score’ was based on a more detailed analysis using the Kuppuswamy scale[1].

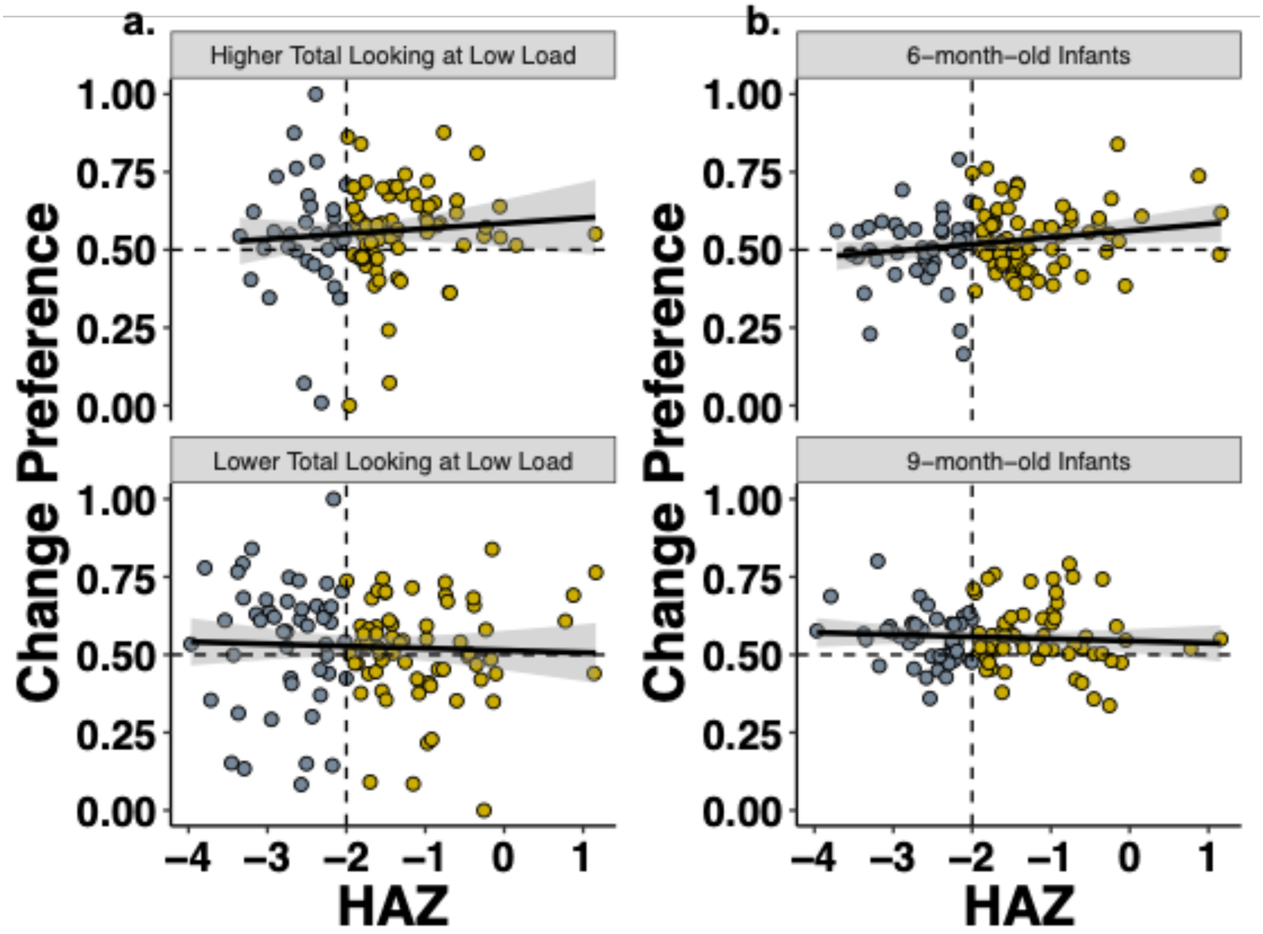

Supplementary Figure 1. (a) Three-way interaction between load, TLT and HAZ. The figure shows data from the low load where the interaction was strongest. Top panel shows data from trials with higher total looking based on a median split; lower panel shows data from trials with lower total looking (based on median split). At the low load, increasing HAZ was associated with increasing CP scores in infants with longer looking durations (top panel). (b) Two-way interaction between age and HAZ, showing that 6-month-olds showed a stronger positive relationship between CP scores and height. In both (a) and (b), colors reflect typical cut-off scores used to identify stunted individuals (z-scores < -2) with stunted infants shown in grey. Across all plots, 0.95 confidence interval is shown in grey.

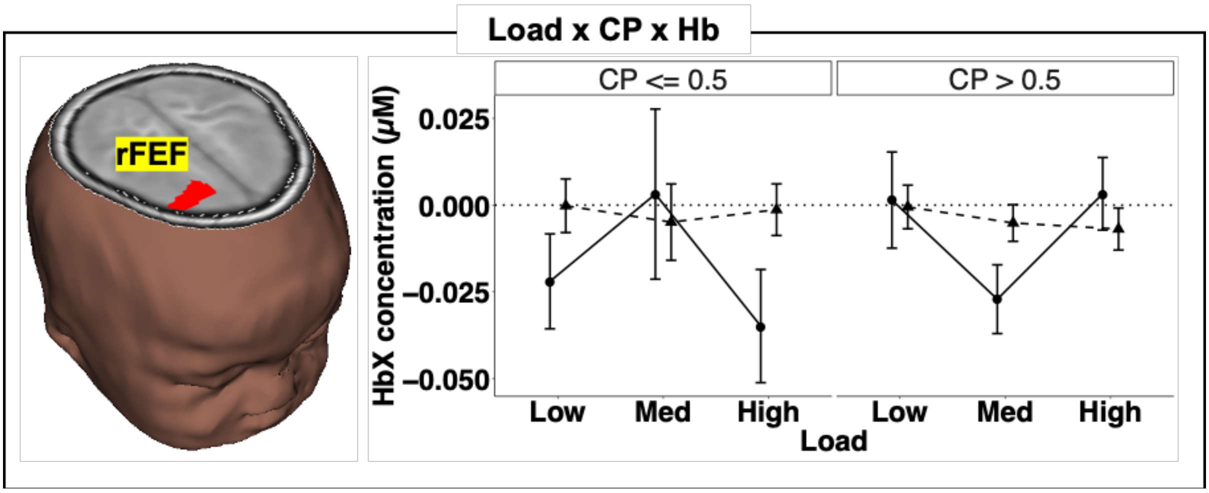

Supplementary Figure 2. Brain image shows location of interaction between load, CP score, and chromophore in right frontal eye fields (rFEF). Right panel shows mean ± SE HbO (solid circles) and HbR (dashed triangles) concentration across the hemodynamic time window (0-20s) for CP scores less then chance (<=0.5) and CP scores greater than chance at the low, medium, and high loads. N = 221 6- and 9-month-old infants.

Assessments carried out in Project INDIA (Infant Neural and Dyadic Interaction Assessment).

- Children's VWM task (behaviour and brain function using fNIRS)
- Dyadic interactions between caregivers and children.
- Standardized assessments (Mullen in year 1 and ASQ in year 2)
- Anthropometry measurements
- Birth history questionnaire (year 1)
- SES/demographic assessment (year 1)
- Early language assessment (year 2)
- Caregiver's VWM task (behaviour only in year 2)
- Domestic violence, depression, and empowerment interview (year 2)
- MRI scan in Lucknow (every 6 months)
- Home assessment during which the following were gathered:
  - 3-days of LENA recordings
  - 3-days of nutrition questionnaires
  - 3-days of sleep diaries
  - 3-days of fitbit recordings
  - Monitoring of in-home air quality
  - Anthropometry measurements

Supplementary analysis of fNIRS data using a channel-based approach.

A key limitation of fNIRS is that optode locations can vary, making it difficult to compare across participants within a study as well as across studies. Spatial variance in brain sampling can occur for many reasons including differences in cap placement on the head and differences in head shape and size. Such spatial variance adds noise to analyses conducted in channel space. To overcome this limitation, there have been key innovations in image-reconstructed fNIRS that uses a head model as a spatial prior to transform channel-based fNIRS data into a volumetric representation [2,3]. We used this approach in the present report. Full details of our approach can be found elsewhere[3].

Although image-based fNIRS analyses are most useful when there are overlapping source-detector pairs (see, e.g., high density diffuse optical tomography[4]), capturing the spatial variance in fNIRS measurements can be impactful even with more typical fNIRS geometries where channels do not overlap. Although a detailed evaluation of this topic is beyond the scope of the current paper, it is useful to examine how a channel-based analysis of our data compares to the image-based analysis from the main report.

Our fNIRS geometry had 36 channels located over frontal, parietal, and temporal cortices (see Figure 1c). To conduct a channel-based analysis, we processed the fNIRS data using Homer2 with the same processing steps described in the methods. Next, we ran an individual-level general linear model using the same 'base' hemodynamic function used in the image-based approach. We also applied the same global signal regression to regress out physiological noise. This created beta values for each chromophore (HbO, HbR) for each load and each participant. We then ran a channel-based group analysis of these data in R using a linear mixed-effects model for each channel separately. As with the image-based analysis, we included fixed effects of load (1, 2, 3), CP score, HAZ score, age, and chromophore as well as a random effect for each participant. To correct for familywise error, we used a significance threshold of $p < .005$.

Based on these analyses, we found 4 channels with significant effects. The locations of these channels are shown in Supplementary Figure 3. To localize these channels, we used an exemplar participant and placed a sphere centered at the location of the maximum sensitivity in cortex from this participant's light model for each channel. Three of these channels overlapped with findings from the main report – rIFG, rFEF, and laIPS – while one effect did not – right superior frontal gyrus. Note that two clusters found in the image-based analysis did not reach significance levels in the channel-based analysis, that is, there were no significant channels near rTPJ and lDLPFC (see Table 1). Overall, this shows robust correspondence between the more traditional channel-based analysis and the image-based results from the main report.

Next, we examined whether the functional effects revealed in the channel-based analysis were comparable to findings from the image-based analysis. We focused on rIFG as this was a key effect that replicated findings from a previous report[5]. Supplementary Figure 4 shows the significant interaction between load, CP score and chromophore for the channel near rIFG (Type III Wald Chi-square = 7.72, p = 0.005). Although there is some variance across loads that was not evident in the image-based analysis, the figure shows that high-performing infants (CP > 0.5) generally showed suppression of rIFG (i.e., negative HbO, positive HbR) consistent with findings in the main report.

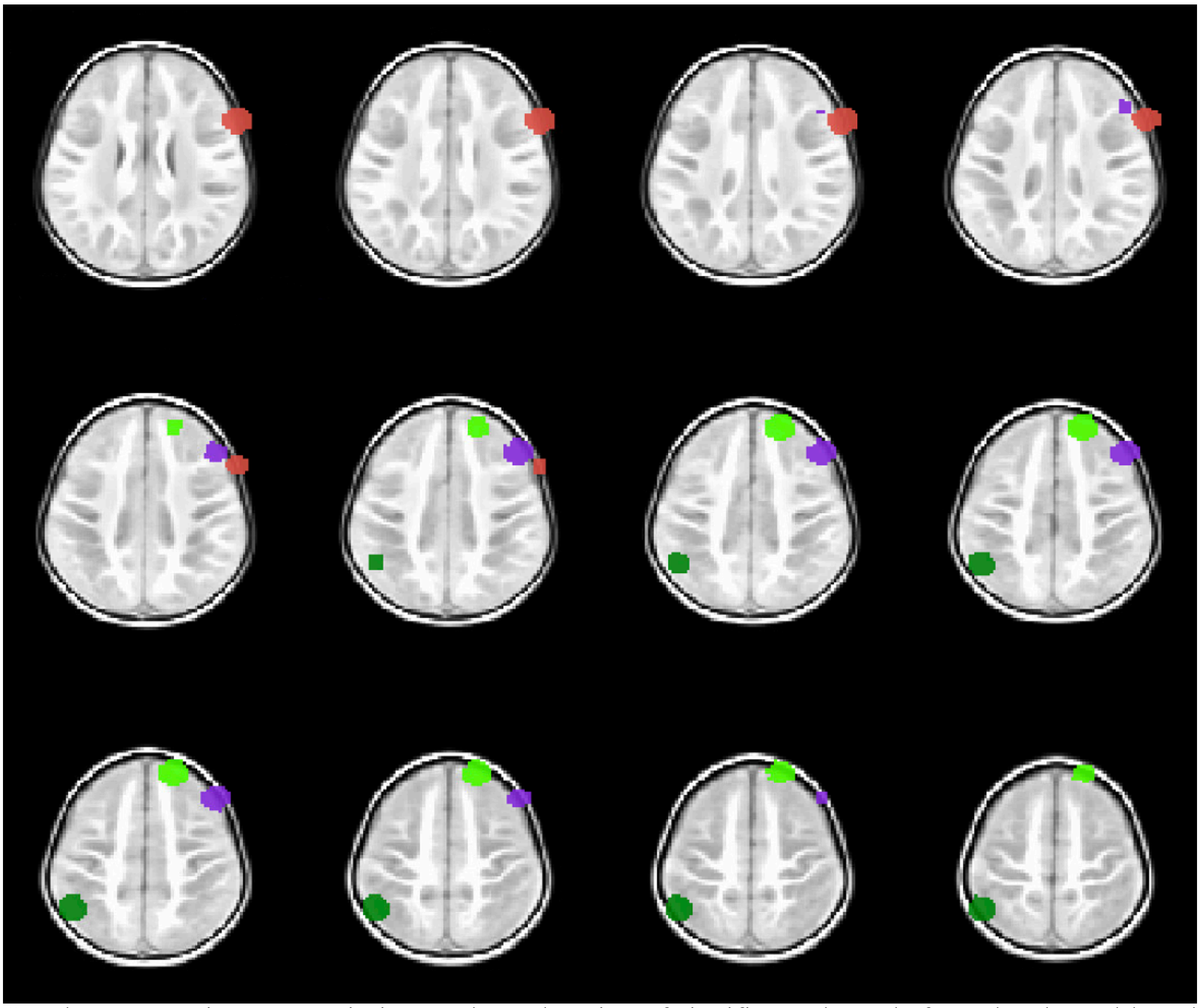

Supplementary Figure 3. Brain image shows location of significant channels from the channel-based analysis. Four channels were significant (p < .005): rIFG (red), right superior frontal gyrus (light green), rFEF (purple), and laIPS (dark green).

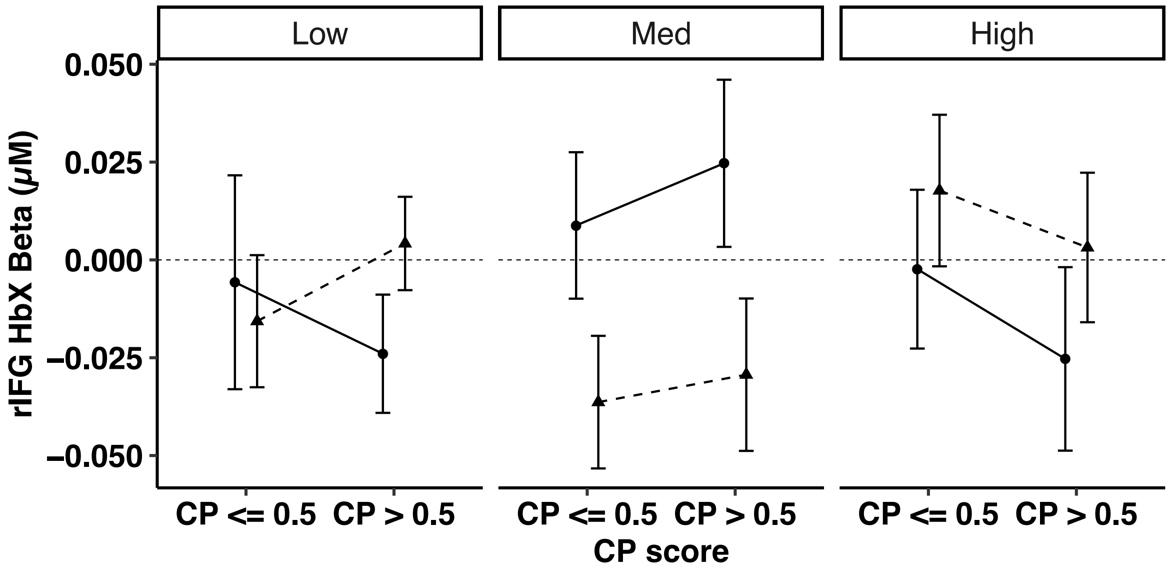

Supplementary Figure 4. Mean ± SE for channel near rIFG showing a significant interaction between load (1, 2, 3), CP score, and chromophore (HbO, HbR). Solid lines show HbO concentrations; dashed lines show HbR concentrations. Distinction along x-axis shows participants with CP <= 0.5 (low) and CP > 0.5 (high). N = 221 6- and 9-month-old infants.

Supplementary analysis of fNIRS data with StdevThresh of 15.

Prior recent studies examining motion correction with infant fNIRS data have recommended that the StdevThresh parameter should be set at 15[6,7]; by contrast, we used a value of 50 in the main text. To examine how this parameter impacted our fNIRS findings, we re-ran all analyses with StdevThresh = 15. For ease of reporting below, we use 'Stdev50' for the analyses reported in the main text and 'Stdev15' for the reanalysis.

Data loss. The first step was to re-process the fNIRS data. As expected, we lost substantially more data following Stdev15. We lost all data from 8 participants. Further, we lost all data from at least one load in 27 participants. We calculated percentage of trials lost across all data (excluding the 8 participants on whom we lost all data) for normal height and stunted infants following Stdev50 and Stdev15 analyses. As can be observed in the table below, for normal height infants, the percentage of trials lost across loads went from under 4% for Stdev50 to about 33% for Stdev15. The percentage of trials lost was more severe for stunted infants; we lost over 40% of their data for Stdev15. This is a very high percentage of data loss for a study that is seeking to understand stunting-related impacts on brain function. Moreover, with high data loss it is likely that the data remaining might not be representative of individual and/or group-level estimates of brain function in at-risk infants.

	Threshold 50 - Low load	Threshold 50 - Medium load	Threshold 50 - High load	Threshold 15 - Low load	Threshold 15 - Medium load	Threshold 15 - High load
**Normal height**	3.9	3.77	3.87	33.95	32.76	33.02
**Stunted**	9.07	8.42	8.12	40.37	42.46	40.76

Supplementary Table 2. Percentage of trials lost for the low, medium, and high load conditions

following Stdev50 and when Stdev15 analyses. Top row shows data loss for normal height infants; bottom row shows data loss for stunted infants.

Spatial alignment. Despite the high data loss, we proceeded with re-running image reconstruction, individual-level GLMs, and finally, group-level linear mixed-effects model (see methods for the details of these steps). We compared spatial alignment of significant clusters from both Stdev50 and Stdev15 approaches. We found substantial alignment between Stdev50 and Stdev15 clusters. All five Stdev50 clusters (see Table 1; main text) could be identified as overlapping, with the same label and/or close to Stdev15 clusters. Supplementary Table 3 shows all significant clusters from the group-level linear mixed effects model following the Stdev15 analysis. In the table, ungraded block colors represent Stdev15 clusters that spatially overlapped with at least one Stdev50 cluster and/or shared the same region label based on distance calculations to specific ROIs described in the Methods (laIPS in blue, lDLPFC in green, and rFEF in orange). Graded colors show Stdev15 clusters that were close to Stdev50 clusters, that is, clusters that were non-overlapping but < 3.7 cm in distance using centre of mass coordinates (rTPJ in yellow – labelled as raIPS; rIFG in grey – labelled as rDLPFC).

**Effect**	**Region of interest**	**Size (mm^3)**	**Centre of mass coordinates**		
			**x**	**y**	**z**
Chromophore	Right ventral occipital complex	319	53.7	-65	125
Load x Chromophore	Left frontal eye fields	289	132.7	-144.1	180.4
CP x Chromophore	Left superior frontal gyrus	563	77.8	-152.3	183.1
CP x Chromophore	Right frontal eye fields (rFEF)	335	64.3	-137.7	184.6
CP x Chromophore	Right anterior intraparietal sulcus	285	48.9	-108.4	179.1
HAZ x Chromophore	Left ventral occipital complex	796	138.4	-58.6	125
HAZ x Chromophore	Left anterior intraparietal sulcus (laIPS)	506	145.6	-77.9	169.7
Age x Chromophore	Left dorsolateral prefrontal cortex (lDLPFC)	365	143	-160.2	164.1
Load x CP x Chromophore	Left anterior intraparietal sulcus (laIPS)	299	146.9	-105.9	182.1
Load x HAZ x Chromophore	Left superior frontal gyrus	402	75.2	-153	181.5
Load x HAZ x Chromophore	Right frontal eye fields (rFEF)	373	66.6	-136.5	182.5
CP x HAZ x Chromophore	Left anterior intraparietal sulcus (laIPS)	574	143.9	-90	181.5
HAZ x Age x Chromophore	Left anterior intraparietal sulcus (laIPS)	301	134.1	-101.8	190.1
Load x CP x HAZ x Chromophore	Right anterior intraparietal sulcus	388	59.8	-92.6	187.6

Load x CP x Age x Chromophore	Left superior frontal gyrus	890	76.3	-153.3	180.8
Load x CP x Age x Chromophore	Left ventral occipital complex	421	142.5	-57	127.8
Load x CP x Age x Chromophore	Left anterior intraparietal sulcus (laIPS)	403	149.4	-104.4	181.7
Load x CP x Age x Chromophore	Left dorsolateral prefrontal cortex (lDLPFC)	278	129.6	-164	175.3
Load x HAZ x Age x Chromophore	Right dorsolateral prefrontal cortex	590	74.2	-161	176.5
Load x HAZ x Age x Chromophore	Left anterior intraparietal sulcus (laIPS)	37	133.4	-96.9	190.6
Load x HAZ x Age x Chromophore	Left dorsolateral prefrontal cortex (lDLPFC)	299	143	-168.3	150.3
CP x HAZ x Age x Chromophore	Right dorsolateral prefrontal cortex	307	75.4	-170.7	168.5
Load x CP x HAZ x Age x Chromophore	Right dorsolateral prefrontal cortex	543	71.4	-170	167
Load x CP x HAZ x Age x Chromophore	Left frontal eye fields	291	132.9	-133.1	185.9

Supplementary Table 3. Significant clusters of brain function following Stdev15 analyses. Effects shown in ungraded block colors represent clusters that either spatially overlapped with at least one Stdev50 cluster and/or shared the same region label based on distance calculations. Effects shown in graded colors represent clusters that were close to Stdev50 clusters (i.e., non-overlapping but < 3.6cm in distance).

Stunting-related effects. In the main text, three regions were associated with stunting status – laIPS, rTPJ and lDLPFC. Following Stdev15 reanalyses, we found that these regions still showed effects of stunting status. A key question is whether the directionality of the Stdev50 and Stdev15 effects were consistent. This was the case: when we examined effects in laIPS, rTPJ, and lDLPFC, we found robust evidence pointing to the same conclusions from the main report. We highlight three representative examples below, two from laIPS and one from lDLPFC.

laIPS. We show overlap between Stdev50 laIPS clusters and Stdev15 laIPS clusters in Supplementary Figure 5a. The effects in these clusters are displayed in line plots in panels (b-e). Panels (b) and (c) correspond with Figure 3a and Figure 4b from the main text (Stdev50 analyses). Panel (d) shows the interaction between HAZ, Age and Chromophore and panel (e) shows the interaction between CP, HAZ and Chromophore – both from Stdev15 analyses. The data in panel (b) show greater engagement of laIPS in 6-month-olds compared to 9-month-olds, reflecting refinement in laIPS recruitment over age (see main text). This same pattern is evident in the Stdev15 results in panel (d), however, there is also a suppression of laIPS activation for 9-month-old stunted infants. This trend is consistent with the main conclusions from our study that stunted infants do not engage laIPS as robustly as normal height infants. As reported in the main text, panel (c) shows robust activity in laIPS for both 6- and 9-month-old normal height infants, with weaker activation for higher-performing infants. By contrast, stunted infants showed weaker activation in this cluster except for higher-performing 6-month-old stunted infants who showed some laIPS engagement. These general patterns are also evident in the Stdev15 results in panel (e), although there are no differences by age. Rather,

higher-performing normal height infants show weaker engagement of laIPS, while higher-performing stunted infants show greater engagement of laIPS. In summary, the overall conclusions regarding laIPS engagement are similar across both analyses.

lDLPFC. We show spatial alignment between Stdev50 and Stdev15 lDLPFC clusters in Supplementary Figure 6a. In panel (b), we show the interaction between HAZ, Age and Chromophore from Figure 5 of the main text. In panel (c), we show the interaction between HAZ, Age, Load and Chromophore from the Stdev15 analyses. In the main text, we noted that DLPFC is often engaged in working memory tasks to support processing in the parietal cortex. Accordingly, panel (b) shows frontal engagement for 6-month-old normal height infants and 9-month-old stunted infants. As can be seen in panel (c), the data from normal height 6-month-olds is very similar; however, the boost in lDLPFC activation is isolated to the medium load. Stunted 9-month-olds also show some evidence of lDLPFC engagement, although only at the high load. Our conclusions following reanalyses largely remain the same. We note that more variability is observed in stunted infants' data following the Stdev15 reanalysis, possibly due to the increase in data loss.

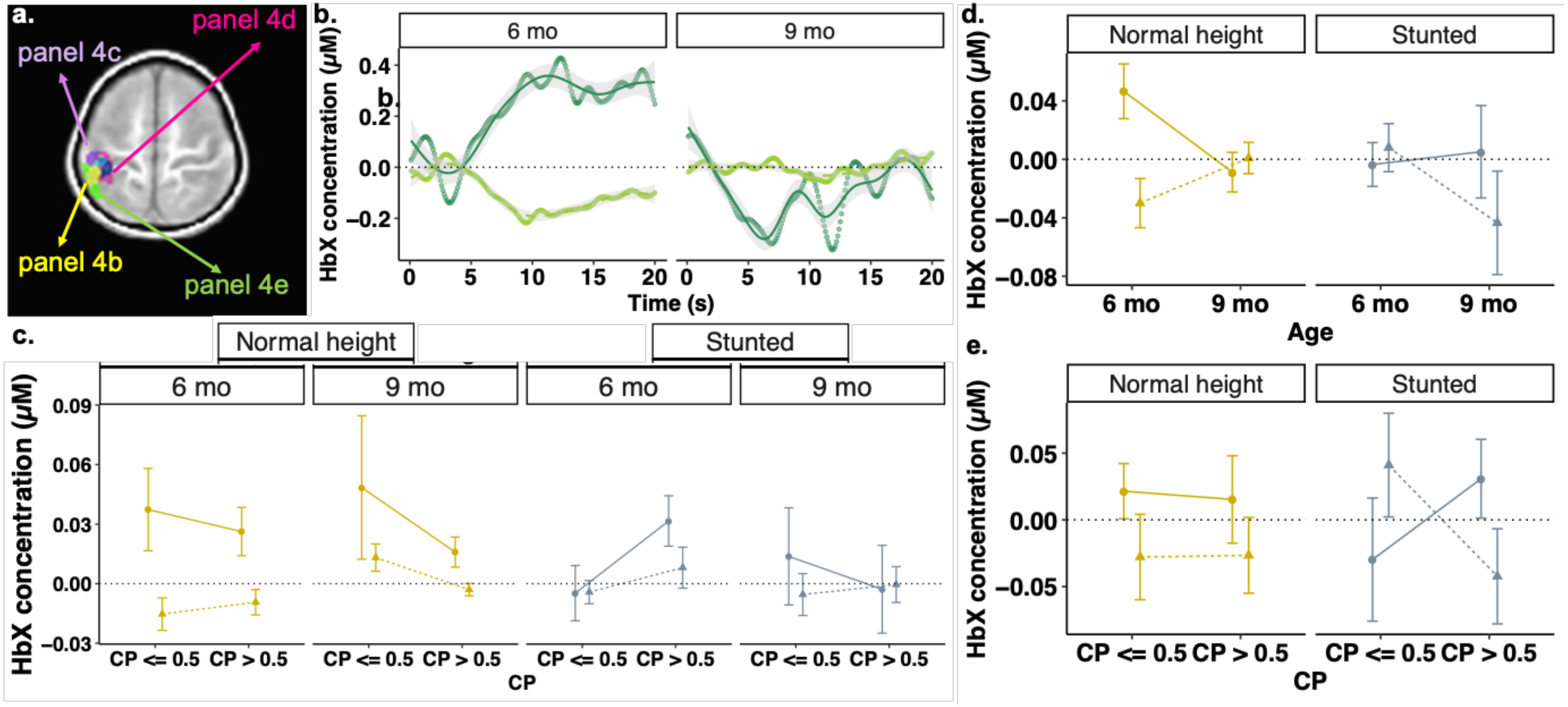

Supplementary Figure 5. (2) Spatial alignment between Stdev50 and Stdev15 laIPS clusters. (b) Figure 3a from main text. (c) Figure 4b from main text. (d) Interaction between HAZ, age and chromophore following Stdev15 analyses. (e) Interaction between CP, HAZ and chromophore following Stdev15 analyses. For time-series plot, the mean is depicted using circle data points; mean smoothed data using a loess function is shown in solid line. Confidence intervals are underlaid and shown in grey. All line plots show mean ± SE. N = 213 6- and 9-month-old infants.

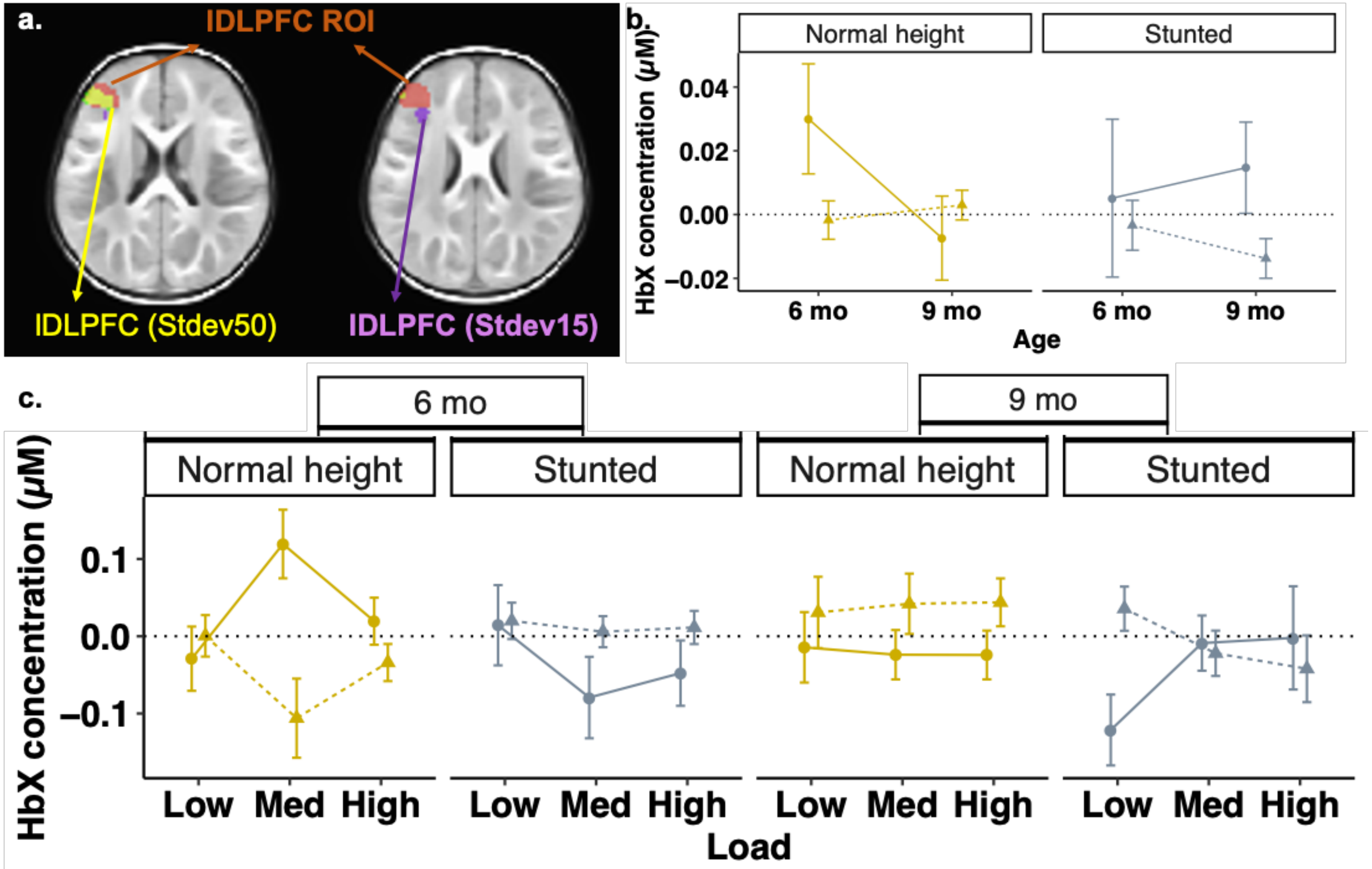

Supplementary Figure 6 (a). Spatial alignment between Stdev50 and Stdev15 lDLPFC clusters. (b) Figure 6 of main text, (c) Interaction between HAZ, Age, Load and Chromophore following Stdev15 analyses. All line plots show mean ± SE. N = 213 6- and 9-month-old infants.

References

1. Shaikh, Z. & Pathak, R. Revised Kuppuswamy and B G Prasad socio-economic scales for 2016. *Int. J. Community Med. Public Heal.* **4**, 997 (2017).
2. Eggebrecht, A. T. *et al.* Mapping distributed brain function and networks with diffuse optical tomography. *Nat. Photonics* **8**, 448–454 (2014).
3. Forbes, S. H. *et al. A processing pipeline for image reconstructed fNIRS analysis using both MRI templates and individual anatomy*. *bioRxiv* (2021).
4. Tripathy, K. *et al.* Decoding visual information from high-density diffuse optical tomography neuroimaging data. *Neuroimage* **226**, 117516 (2021).
5. Wijeakumar, S., Kumar, A., M. Delgado Reyes, L., Tiwari, M. & Spencer, J. P. Early adversity in rural India impacts the brain networks underlying visual working memory. *Dev. Sci.* (2019) doi:10.1111/desc.12822.
6. Di Lorenzo, R. *et al.* Recommendations for motion correction of infant fNIRS data applicable to multiple data sets and acquisition systems. *Neuroimage* **200**, 511–527 (2019).
7. Gemignani, J. & Gervain, J. Comparing different pre-processing routines for infant fNIRS data. *Dev. Cogn. Neurosci.* **48**, 100943 (2021).
