# Supplementary material for: Stunting in infancy is associated with atypical activation of working memory and attention networks
Source: Nat Hum Behav. 2023 Oct 26;7(12):2199–211. doi: 10.1038/s41562-023-01725-3 (PMC10730391; doi:10.1038/s41562-023-01725-3)
Supplement: Supplementary file 2 — Reporting Summary [file 41562_2023_1725_MOESM2_ESM.pdf]

nature portfolio

Corresponding author(s): Sobanwartiny Wijeakumar and John Spencer

Last updated by author(s): Aug 31, 2023

Reporting Summary

Nature Portfolio wishes to improve the reproducibility of the work that we publish. This form provides structure for consistency and transparency in reporting. For further information on Nature Portfolio policies, see our Editorial Policies and the Editorial Policy Checklist.

Statistics

For all statistical analyses, confirm that the following items are present in the figure legend, table legend, main text, or Methods section.

n/a	Confirmed	
☐	☒	The exact sample size (*n*) for each experimental group/condition, given as a discrete number and unit of measurement
☐	☒	A statement on whether measurements were taken from distinct samples or whether the same sample was measured repeatedly
☐	☒	The statistical test(s) used AND whether they are one- or two-sided<br>*Only common tests should be described solely by name; describe more complex techniques in the Methods section.*
☐	☒	A description of all covariates tested
☐	☒	A description of any assumptions or corrections, such as tests of normality and adjustment for multiple comparisons
☐	☒	A full description of the statistical parameters including central tendency (e.g. means) or other basic estimates (e.g. regression coefficient) AND variation (e.g. standard deviation) or associated estimates of uncertainty (e.g. confidence intervals)
☐	☒	For null hypothesis testing, the test statistic (e.g. F, t, r) with confidence intervals, effect sizes, degrees of freedom and P value noted<br>*Give P values as exact values whenever suitable.*
☒	☐	For Bayesian analysis, information on the choice of priors and Markov chain Monte Carlo settings
☐	☒	For hierarchical and complex designs, identification of the appropriate level for tests and full reporting of outcomes
☐	☒	Estimates of effect sizes (e.g. Cohen's d, Pearson's r), indicating how they were calculated

Our web collection on statistics for biologists contains articles on many of the points above.

Software and code

Policy information about availability of computer code

Data collection	Experiment Builder (SR Research) was used to present the experimental task and collect eye-tracking dat from the infant. fNIRS data was collected using TechEn CW7 software.
Data analysis	Eye-movement data from the eye-tracker and video recordings were analyzed using the package eyetrackingR and open software datavyu. Head models were generated from MRI data using AFNI v2 and ANTS v2.1. fNIRS data was analyzed using a pipeline of custom-written code in R, MATLAB (HOMER v2 package) and AFNI v2. Group-level behavioral and brain imaging analyses was conducted using custom-written code in AFNI v2 and R.

For manuscripts utilizing custom algorithms or software that are central to the research but not yet described in published literature, software must be made available to editors and reviewers. We strongly encourage code deposition in a community repository (e.g. GitHub). See the Nature Portfolio guidelines for submitting code & software for further information.

Data

Policy information about availability of data

All manuscripts must include a data availability statement. This statement should provide the following information, where applicable:

- Accession codes, unique identifiers, or web links for publicly available datasets
- A description of any restrictions on data availability
- For clinical datasets or third party data, please ensure that the statement adheres to our policy

Data availability
All behavioural and brain data used in statistical analyses, including scripts and code are publicly available on doi.org/10.17605/OSF.IO/KC3N8.

Human research participants

Policy information about studies involving human research participants and Sex and Gender in Research.

Reporting on sex and gender	Sex distribution is reported in supplementary table 1. Sex was included as a covariate in preliminary analyses but did not contribute significantly to statistical models and was therefore dropped from final analyses.
Population characteristics	The population characteristics are described in detail in supplementary table 1.
Recruitment	Families with infants aged 6 months ⍰ 15 days or 9 months ⍰ 15 days from the villages in and around Shivgarh in the district of Raebarelli, Uttar Pradesh, India were contacted by researchers from the Community Empowerment Lab (CEL). Infants born to parents screened with colour vision deficits (due to the nature of the VWM task) or any congenital problems, or gestational age < 26 weeks at birth were excluded from the study. Infants were enrolled across four waves of data collection separated by 3 months from May 2017 to February 2018 (year 1). Approximately 30 6-month-olds and 30 9-month-olds were enrolled in each wave. Infants were also followed up for another year from 2018 to 2019 (year 2).
Ethics oversight	Approval for this study was provided by the Institutional Ethics Committee at the Community Empowerment Lab (CELIEC/2017002), Lucknow, India.

Note that full information on the approval of the study protocol must also be provided in the manuscript.

Field-specific reporting

Please select the one below that is the best fit for your research. If you are not sure, read the appropriate sections before making your selection.

☐ Life sciences ☒ Behavioural & social sciences ☐ Ecological, evolutionary & environmental sciences

For a reference copy of the document with all sections, see nature.com/documents/nr-reporting-summary-flat.pdf

Behavioural & social sciences study design

All studies must disclose on these points even when the disclosure is negative.

Study description	The study is quantitative experimental.
Research sample	The sample consisted of 6 and 9 month-old infants from rural India (Shivgarh in Uttar Pradesh) followed across two years. Demographic information has been provided in Supplementary table 1. The rationale for choosing this sample of participants was to investigate the impact of prevalence of stunting on neurocognitive development. The chosen sample is representative.
Sampling strategy	All known families with infants aged 6 months +/- 15 days or 9 months +/- 15 days from the villages in and around Shivgarh in the district of Raebarelli, Uttar Pradesh, India were contacted by researchers from the Community Empowerment Lab (CEL). Families who were interested in the study and met the inclusion criteria were invited to visit the lab. Infants born to parents screened with colour vision deficits (due to the nature of the VWM task) or any congenital problems, or gestational age < 26 weeks at birth were excluded from the study.
Data collection	General procedures for the laboratory visit. Families were transported in groups from their homes to the CEL Facility in Shivgarh. Three researchers were present during the sessions. The researchers were not blinded to the experimental condition and study hypothesis. The family were first escorted to the waiting room of the facility. Some groups of families were also provided a tour of the facility and a demonstration of the procedures to make them feel more comfortable and allow them to ask questions. Next, the families were escorted back to the waiting room where informed consent was sought. Participants' caregivers provided written informed consent; where caregivers were illiterate, a witness gave signed consent accompanied by a thumb impression of the caregiver in place of a signature. After consent was obtained, physical measurements of the infant were taken. The infant's head

circumference was also measured to prepare an appropriately sized cap for fNIRS data collection.
Next, the parent and the infant were escorted to the fNIRS assessment room. The room was colored in a neutral grey to prevent distraction of the infant. The mother was seated on a chair and the infant was placed on the mother's lap approximately 100 cm away from the TV screen (see Figure 1b). A cartoon was played on a TV screen to engage the infant. When the infant looked comfortable, two researchers placed an appropriately sized fNIRS cap on the head and fastened the chinstrap to hold it in place. After adjusting fNIRS signals (e.g., clearing hair from under individual fNIRS optodes), one researcher proceeded to use a Polhemus sensor to collect coordinates of the scalp landmarks and source and detector positions, while a second researcher placed a calibration sticker on the infant's forehead and set up the eye-tracker to record eye-movements from the infant. A 5-point calibration sequence was played on the monitor to ensure correct eye-tracking at the top, bottom, left, right and central parts of the screen. After this, the VWM task was presented and video recordings, eye-tracking and fNIRS data collection ensued. If the infant showed signs of distress, cartoon clips were played in-between trials. Breaks were provided if the infant needed to be fed, fell asleep, or could not be calmed even after the use of the cartoon clips. The family was escorted back to the waiting room after the completion of the assessment or if the infant and/or mother needed a break from the assessment. In year 2, the mother and infant were escorted to another room to administer the ASQ assessment. At the end of each laboratory session, families received a small gift for participating in the study.

Physical growth measurements. Physical growth measurements were taken during laboratory visits, home visits, and MRI visits unless two or more visits were close in time, in which case, a single measurement was used for that time point. Measurements of head circumference, mid-upper arm circumference, calf circumference, infant's weight, and infant's length were taken by two members of the research team who were trained through a standardized workshop. Head circumference, mid-upper arm circumference, and calf circumference were measured twice using a SECA measurement tape. Measurements were repeated if there were any discrepancies of > 7 mm between two measurements. An infantometer was used for measuring the child's length from head to heel with 1 mm precision. A digital SECA weighing scale was used to measure the baby's weight with 10 grams precision.

VWM task. Infants were presented with a preferential looking VWM task15 during the laboratory visit. A PC running Experiment Builder (SR Research) was used to present the task on a 42-inch LCD TV screen. Infants sat on their parents' lap approximately 100 cm away from the screen. Each stimulus display area was 29.5cm in width and 21cm in height, with a 21cm gap between the display on the left and right (each colored square was approximately 5cm x 5cm). The displays had a solid grey background. The colors of the squares presented on each display were selected from a set of nine colors: green (RGB: 0, 153, 0), brown (128, 64, 32), black (0, 0, 0), violet (128, 0, 128), cyan (128, 255, 255), yellow (255, 255, 0), blue (0, 0, 255), white (255, 255, 255), and red (255, 0, 0). On a display, the colors of the squares differed from each other, but colors could be repeated between the displays (i.e., the same color could appear on both displays). The positions of the squares on each display were randomly selected from a 3-by-3 grid of possible positions. Eye-movement data was recorded using an Eyelink 1000 Plus eye-tracker (SR research) operating in binocular mode with a sampling rate of 500 Hz. Additionally, one camera recorded a view of the infants' face, and another camera recorded the TV display. These video recordings were used to extract looking data when eye-tracking information was not available (due to technical problems, reflectance, poor lighting, loss of calibration).
Each trial started with a dynamic attention cue. Once the eye-tracker / experimenter detected that the infant was looking at the attention-getter, the task proceeded to the VWM displays. Each trial consisted of side-by-side displays of colored squares that appeared for 500 ms and disappeared for 250 ms for a trial duration of 10 seconds. Each trial was followed by a minimal inter-trial interval of 5s, however, this period was typically longer as the trial was not initiated until the infant looked at the display following the dynamic attention cue. On the 'unchanging' side, the colors of the squares remained the same across each flash, while on the 'changing' side, one square changed its color across each flash. Visual working memory load was manipulated by varying the number of squares on each side across trials (1, 2, or 3 squares on each side). The aim was to present each infant with 36 total trials in six blocks of 6 trials, although where the infant and parent were willing to continue, additional blocks were sometimes collected. Each block contained 2 trials for each load, one with the changing side on the left, one with the changing side on the right. Order of trials was randomized in each block. Where necessary, participants could take a break between blocks.

Functional near-infrared spectroscopy (fNIRS) data acquisition. fNIRS data was collected from infants as they engaged with the VWM task during the laboratory visit. A TechEn CW7 system and software (12 sources and 24 detectors) with wavelengths of 830 nm and 690 nm and sampling rate of 25 Hz was used to collect brain function data. Fiber optic cables were used to carry light from the TechEn system to a cap with a customized probe geometry of 36 channels overlying the frontal, parietal, and temporal cortices (see Figure 1c). A laptop connected to the fNIRS system recorded and displayed data as it was being collected. This laptop was also connected to the Experiment Builder computer to synchronize fNIRS data with the start of each trial of the task. A Polhemus Patriot Motion Sensor was used to digitize scalp landmarks and positions of sources and detectors on the cap.

MRI data acquisition. Anatomical data were collected on a Philips Achieva 3T MRI scanner equipped with 12-channel head RF array in an MRI Facility in Lucknow, India. The protocol used volumetric T1-weighted SPGR acquisition. All imaging was performed during natural sleep49. Acquisition parameters were as follows: For T1 SPGR: Field of View (FoV) = 19 x 19cm; slice thickness (ST) = 1.2 mm; acquisition matrix = 194 x 194; flip angle = 9º; echo time (TE) = 3.72 ms; repetition time (TR) = 9.5 ms; and receiver bandwidth (BW) = 270 Hz/voxel.

Ages-and-Stage Questionnaire III (ASQ) assessment. The ASQ was administered during laboratory visits in year 2 when the infants were 18 months (for the 6-month-old cohort) or 21 months (for the 9-month-old cohort). The appropriate ASQ questionnaire for each infant was selected using the online ASQ calculator (https://agesandstages.com/free-resources/asq-calculator/). While ASQ is designed as a screening questionnaire to be completed by parents, we adapted its administration to improve the reliability of the data. Specifically, a trained assessor administered the ASQ in collaboration with the parent. In cases where questions from the ASQ materials kit asked about behaviors that could be elicited in the laboratory (e.g., 'When you ask your child to, does he go into another room to find a familiar toy or object?'), these tasks were completed 'live', ensuring that the child was given ample time to perform each task. In the event the child was unable to perform the task, or the question was not amenable to live assessment, the mother's verbal report on the question was taken as the response. The ASQ yields five subscales of development: communication, gross motor, fine motor, problem-solving, and personal-social. Each subscale contained 6 questions, making up a total of 30 questions on the form. For this study, we focused on the problem-solving scale as it was most directly related to VWM function, and we were interested in investigating later cognitive outcomes.

Timing

Infants were enrolled across four waves of data collection separated by 3 months from May 2017 to February 2018 (year 1).

Timing	Approximately 30 6-month-olds and 30 9-month-olds were enrolled in each wave. Infants were also followed up for another year from 2018 to 2019 (year 2).
Data exclusions	Data from 17 infants were excluded from all analyses due to problems with the behavioural and neuroimaging data collection and processing (not enough behavioural data in 9 infants, technical problems with the neuroimaging system for 7 infants, and neuroimaging data lost due to motion artifacts from 1 infant).
Non-participation	277 families met the inclusion criteria and gave due consent. The study had no provision for compensation; however, the participant families were offered a gift hamper with baby items as a token of appreciation. From this sample, 37 children did not complete the first in-take assessment (19 6-month-olds and 17 9-month-olds). The remaining 240 families were enrolled into the study.
Randomization	Participants were not allocated into response groups.

Reporting for specific materials, systems and methods

We require information from authors about some types of materials, experimental systems and methods used in many studies. Here, indicate whether each material, system or method listed is relevant to your study. If you are not sure if a list item applies to your research, read the appropriate section before selecting a response.

Materials & experimental systems

n/a	Involved in the study	
☒	☐	Antibodies
☒	☐	Eukaryotic cell lines
☒	☐	Palaeontology and archaeology
☒	☐	Animals and other organisms
☒	☐	Clinical data
☒	☐	Dual use research of concern

Methods

n/a	Involved in the study	
☒	☐	ChIP-seq
☒	☐	Flow cytometry
☐	☒	MRI-based neuroimaging

Magnetic resonance imaging

Experimental design

Design type	Not applicable.
Design specifications	Not applicable.
Behavioral performance measures	Not applicable.

Acquisition

Imaging type(s)	Structural
Field strength	3T
Sequence & imaging parameters	Anatomical data were collected on a Philips Achieva 3T MRI scanner equipped with 12-channel head RF array in an MRI Facility in Lucknow, India. The protocol used volumetric T1-weighted SPGR acquisition. All imaging was performed during natural sleep49. Acquisition parameters were as follows: For T1 SPGR: Field of View (FoV) = 19 x 19cm; slice thickness (ST) = 1.2 mm; acquisition matrix = 194 x 194; flip angle = 9º; echo time (TE) = 3.72 ms; repetition time (TR) = 9.5 ms; and receiver bandwidth (BW) = 270 Hz/voxel.
Area of acquisition	Whole brain

Diffusion MRI ☐ Used ☒ Not used

Preprocessing

Preprocessing software	The starting point for the processing pipeline is an anatomical T1-weighted image that approximately has the nose aligned with the y axis of the scanner [i.e., the nose should be pointing forward (anterior) in an axial image]. If the orientation of the nose is more than 15 deg from this orientation—for example in a sleeping infant—we have found it helpful to rotate the image such that the nose is roughly aligned with the y-axis for initiating the pipeline. This can be done using 3drotate from AFNI or similar functionality from other image analysis packages. Next, the image is resampled (3dresample) into a standard right-axial-superior orientation. This ensures that the image will be oriented properly once imported into AtlasViewer. The next step of the segmentation pipeline is bias field correction (3dUnifize); this is optional and can be used if large variations in the signal exist across the image.We then create a brain mask to define brain tissue in the image. This step is sensitive to the age of the subject being analyzed since infants have a significantly smaller

intracranial volume as compared to adults. Thus, this step has two options to generate the brain mask: 3dSkullstrip and ROBEX.20 The 3dSkullstrip option performs well in most cases and the user is provided with the ability to set the initial radius and expansion rate for the sphere inflation used in the underlying algorithm. In infant scans, we have found improved performance with the ROBEX-based brain mask. For details on using these functions, we can see the instruction guide available at https://github.com/developmentaldynamicslab/MRI-NIRS_Pipeline.

The next step of the pipeline is to put the T1 weighted scan into anterior commissure (AC)-posterior commissure (PC) alignment. To do this, the brain mask in the prior step is used to extract the brain from the T1-weighted image, which is subsequently aligned with a skullstripped Talairach Atlas image using auto_tlrc command from AFNI. The rigid body transform from the resulting transform is saved and used to reorient the subject T1-weighted scan and brain mask into AC-PC alignment. Next, we pad the image with zeros. This ensures that the surface generated will be closed when imported into AtlasViewer. We then generate a skull mask from the image by identifying an optimal threshold (3dClipLevel) for background removal. The resulting mask generated from thresholding at this optimal level is then filled to define all voxels within the skull. The next step of the pipeline is optional and median filters the image. This may be necessary for low SNR images. The final step of the pipeline uses the 3dSeg command from AFNI to define the tissue into gray matter, white matter, and CSF. The resulting segmentation is then combined with the skull segmentation to generate a label map that contains four labels: gray matter, white matter, CSF, and skull.

Normalization

Not applicable.

Normalization template

To create a head model for each infant, we used the anatomical MRI scan if it was available. Out of the 223 children included in the analyses, anatomical T1-weighted images were available for 72 6-month-old infants and 70 9-month-old infants. The remaining infants did not have an anatomical scan (45 6-month-olds and 36 9-month-olds). If a scan was not available, we used an age-specific MRI template. A 6-month-old template and a 9-month-old template were created from the available scans of 15 boys and 15 girls for each specific age, using a multistep registration procedure. This procedure was carried out using antsMultivariateTemplateConstruction provided by ANTS 2.1. Briefly, all the images were linearly aligned and averaged to provide a template estimate. Then, all images were nonlinearly aligned to this initial estimate. The results were averaged to provide an improved estimate. This process was repeated ten times to construct the final estimate. The 6-month-old template was used for the 45 6-month-olds who did not have anatomical scans and the 9-month-old template was used for the 36 9-month-old infants who did not have anatomical scans.

Noise and artifact removal

Not applicable.

Volume censoring

Not applicable.

Statistical modeling & inference

Model type and settings

Not applicable.

Effect(s) tested

Not applicable.

Specify type of analysis: ☐ Whole brain ☐ ROI-based ☐ Both

Statistic type for inference
(See Eklund et al. 2016)

Not applicable.

Correction

Not applicable.

Models & analysis

n/a	Involved in the study
☒	☐ Functional and/or effective connectivity
☒	☐ Graph analysis
☒	☐ Multivariate modeling or predictive analysis
